# Equilibrium oxygen storage capacity of ultrathin CeO_2-δ_ depends non-monotonically on large biaxial strain

**DOI:** 10.1038/ncomms15360

**Published:** 2017-05-18

**Authors:** Chirranjeevi Balaji Gopal, Max García-Melchor, Sang Chul Lee, Yezhou Shi, Andrey Shavorskiy, Matteo Monti, Zixuan Guan, Robert Sinclair, Hendrik Bluhm, Aleksandra Vojvodic, William C. Chueh

**Affiliations:** 1 Department of Materials Science and Engineering, Stanford University, Stanford, California 94305, USA; 2 SUNCAT Center for Interface Science and Catalysis, SLAC National Accelerator Laboratory, Menlo Park, California 94025, USA; 3 Department of Chemical Engineering, Stanford University, Stanford, California 94305, USA; 4 School of Chemistry, Trinity College Dublin, College Green, Dublin 2, Ireland; 5 MAX IV Laboratory, Lund University, 225 94 Lund, Sweden; 6 Department of Applied Physics, Stanford University, Stanford, California 94305, USA; 7 Chemical Sciences Division, Lawrence Berkeley National Laboratory, Berkeley, California 94720, USA; 8 Department of Chemical and Biomolecular Engineering, University of Pennsylvania, Philadelphia, Pennsylvania 19104, USA; 9 Stanford Institute for Materials and Energy Sciences, SLAC National Accelerator Laboratory, Menlo Park, California 94025, USA

## Abstract

Elastic strain is being increasingly employed to enhance the catalytic properties of mixed ion–electron conducting oxides. However, its effect on oxygen storage capacity is not well established. Here, we fabricate ultrathin, coherently strained films of CeO_2-δ_ between 5.6% biaxial compression and 2.1% tension. *In situ* ambient pressure X-ray photoelectron spectroscopy reveals up to a fourfold enhancement in equilibrium oxygen storage capacity under both compression and tension. This non-monotonic variation with strain departs from the conventional wisdom based on a chemical expansion dominated behaviour. Through depth profiling, film thickness variations and a coupled photoemission–thermodynamic analysis of space-charge effects, we show that the enhanced reducibility is not dominated by interfacial effects. On the basis of *ab initio* calculations of oxygen vacancy formation incorporating defect interactions and vibrational contributions, we suggest that the non-monotonicity arises from the tetragonal distortion under large biaxial strain. These results may guide the rational engineering of multilayer and core–shell oxide nanomaterials.

## Abstract (web summary)

The surface oxygen storage capacity is an important metric of catalytic activity, but its dependence on strain is not well characterized. Here, the authors show the surface oxygen nonstoichiometry in coherently strained CeO2-δ films increases non-monotonically with biaxial strain.

The tunable oxygen nonstoichiometry of mixed ionic and electronic conducting (MIEC) oxides underlies their application in solid oxide fuel cell electrodes^1^, heterogeneous catalysis^2^, solar fuel generation through thermochemical cycles^3^ and resistive switching-based memory devices^4^. It governs the concentration of mobile oxygen vacancies and electronic defects that mediate oxygen exchange at the oxide/gas interface, store oxygen at the surface and in the bulk and facilitate transport^5^. The oxygen nonstoichiometry can be tuned intrinsically through temperature and oxygen partial pressure, and extrinsically through composition, nanostructuring and lattice strain. Recent advances in achieving atomic-level control in thin-film growth techniques, the inherent layered architecture of solid-state devices and their rapid downsizing have rendered lattice strain an attractive and dopant-free alternative to enhance a wide array of properties of existing materials^6^. Strain-induced modifications to the crystal symmetry of multilayered thin films and core–shell nanomaterials have led to dramatic enhancements in carrier mobilities of semiconductors^7^, superconducting transition temperatures^8^,^9^ and ferroic properties of layered oxides^10^,^11^.

In MIEC oxides, strain has an additional impact on the material properties through its coupling with oxygen chemical potential. The presence of oxygen vacancies is associated with an expansion of the lattice due to electron localization on the neighbouring cations, commonly known as chemical expansion. Thus, control of lattice volume through strain provides a means to tune the defect chemical properties of MIEC oxides. Numerous studies have investigated the effect of strain on the surface reactivity using substrate-supported films^12^,^13^,^14^,^15^ and on ionic transport using multilayered heterostructures^16^,^17^,^18^,^19^,^20^,^21^,^22^,^23^. In the latter, a wide variation in the results is often observed depending on the volume fraction of the strained phases and the geometry of electrical contacts, among other parameters^20^,^21^,^22^,^23^,^24^. It is generally recognized that compressive strain increases the migration barrier for oxygen ion transport and surface exchange, while tensile strain has the opposite effect. This hypothesis of a monotonic dependence of reaction barriers on strain has been corroborated by computational studies^25^,^26^,^27^, and is believed to extend to oxygen vacancy formation energetics as well^28^,^29^,^30^. Such a trend is consistent with a chemical expansion-dominated behaviour^31^. However, strain also affects the ligand field and distorts local bonding which can influence the vacancy formation and migration significantly^32^. Yet another important consideration for MIECs with large point defect concentrations is the chemical strain effect that describes the alleviation of elastic strain energy through defect association and formation of concentration gradients^33^,^34^,^35^. In Gd-doped ceria, for instance, defect association lowers the apparent elastic modulus by over an order of magnitude to accommodate large stresses^34^. Decoupling the multiple effects of strain necessitates well-defined model systems and precise fabrication control, as elegantly pointed out by Yildiz^36^ and Wen *et al*.^37^ in recent reviews.

An important metric of catalytic activity of MIEC oxides is the surface redox capacity, that is, the extent of change in oxygen nonstoichiometry with oxygen chemical potential. Changes to the surface chemistry and its ability to buffer oxygen can have dramatic implications for both the thermodynamics and kinetics of oxygen insertion reactions. To establish a definitive relationship between strain and a functional property, it is desirable to have the oxide film uniformly strained over a wide range of values, both compressive and tensile. This is challenging, because biaxially strained films undergo strain relaxation beyond a critical film thickness, rendering the strain fields inhomogeneous^38^, or undergo a polymorphic transition due to large local strains^39^. For instance, in a recent study using electron energy-loss spectroscopy to probe the incoherent CeO_2-δ_/YSZ interface, Song *et al*.^38^ found that the Ce atoms are reduced to the 3+ oxidation state near the misfit dislocation, regardless of the sign of the strain. However, Sun *et al*.^30^ used atomistic simulations and arrived at an opposite prediction. Donner *et al*.^40^ showed that strained La_0.5_Sr_0.5_CoO_3–δ_ thin films undergo cation ordering (unlike the bulk oxide), facilitated by lower oxygen vacancy formation energy and enhanced cation mobility under biaxial strain. Another recent *ex situ* study of strained SrCoO_3-δ_ observed an increase in oxygen nonstoichiometry with tensile strain^41^, though the non-equilibrium nature of the experiment convolutes the kinetics and thermodynamics of oxygen vacancy formation. As can be seen, the difficulty of isolating strain effect from that of the buried interface and misfit dislocations, and the lack of *in situ* measurements, have precluded a clear connection between biaxial strain and oxygen storage capacity.

In this study, we quantify the effect of large biaxial strain on the surface redox capacity of CeO_2-δ_, a prototypical MIEC with wide-ranging applications in chemical catalysis and electrocatalysis^2^. Ceria shows exceptional oxygen nonstoichiometry and phase stability in its bulk form over a range of reducing conditions and elevated temperatures, making it an ideal model system. Owing to the immense lattice mismatch between ceria and YSZ (5.6%), a coherent interface between the two has been regarded as impossible to stabilize. In fact, equilibrium theory predicts a critical thickness of ∼0.8 nm (<2 unit cells, see Supplementary Note 1)^42^. We successfully fabricate ultrathin cerium oxide films under biaxial compression on atomically flat, single-crystalline (001) YSZ^43^, and under biaxial tension on (001) STO. Using *in situ* ambient pressure X-ray photoelectron spectroscopy (APXPS), we directly quantify the surface Ce^3+^ and oxygen vacancy concentrations of the strained ceria films under conditions relevant to solid oxide fuel cells and catalysis, and compare them with those of fully relaxed films. Remarkably, both compressive and tensile strained films showed a significant enhancement in the Ce^3+^ and oxygen vacancy concentrations near the surface compared with the unstrained films. Using depth profiled APXPS and by varying the thickness of strained films, we show that the buried interface does not contribute significantly to the observed strain effects. The use of coherently strained films (−5.6% and 2.1%) and measuring oxygen nonstoichiometry in chemical equilibrium with the gas phase capture the true effect of strain. This reproducible, non-monotonic behaviour goes against the conventional wisdom that defect chemical properties vary monotonically with lattice strain and opens up new means to engineer enhanced redox capacity in multilayer structures. By combining APXPS, coupled photoemission–space-charge analysis and density functional theory (DFT) calculations, we demonstrate a systematic framework for understanding and isolating the various thermodynamic contributions (apart from strain) to defect formation at the surface.

## Results

### Characterization of strain and interface structure

**Figure 1:**
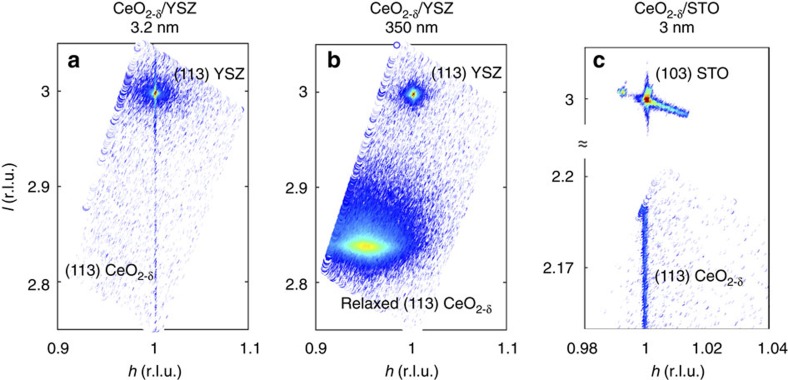
-ray reciprocal space maps of coherently strained versus relaxed ceria. RSM about the (113) reflection of YSZ for (**a**) 3.2 nm ceria on YSZ (compressively strained) (**b**) 350 nm ceria on YSZ (unstrained/relaxed) and (**c**) about the (103) reflection of STO for the 3 nm ceria on STO (tensile strained). For the strained oxides, that is, (**a**,**c**), the film peak shows up directly below that of the substrate (same ‘*h*'), confirming identical in-plane lattice constants, that is, coherency. For the relaxed film, the ceria peak position corresponds to its equilibrium lattice constant; r.l.u., reciprocal lattice unit.

Three sets of CeO_2-δ_ films were studied: (1) 3.2 nm thick, compressively strained on (001) YSZ, (2) 350 nm thick, unstrained on (001) YSZ and (3) 3.0 nm thick, tensile strained on (001) STO. The strain state of the ceria films was quantified using X-ray reciprocal space mapping (RSM). For CeO_2-δ_/YSZ, Fig. 1a shows the RSM about the off-specular (113) reflection. The sharp, intense feature centred at (113) is that of the substrate. The faint (113) ceria reflection immediately below the substrate reflection indicates that its in-plane lattice constant is matched to that of YSZ. From the absence of any intensity at approximately (0.95 0.95 2.85) corresponding to relaxed ceria (Fig. 1b), and diffuse features in between, we conclude that the film is coherently strained. For CeO_2-δ_/STO, RSM was performed about the STO (103) and ceria (113) reflections, because of the 45° in-plane rotation for registry alignment (see Methods). As evident in Fig. 1c, the in-plane reciprocal lattice vectors of the film and substrate are once again matched, and the relaxed ceria feature expected at (2.15 2.15 1.02) is absent, confirming that ceria is coherently strained on STO as well.

**Figure 2:**
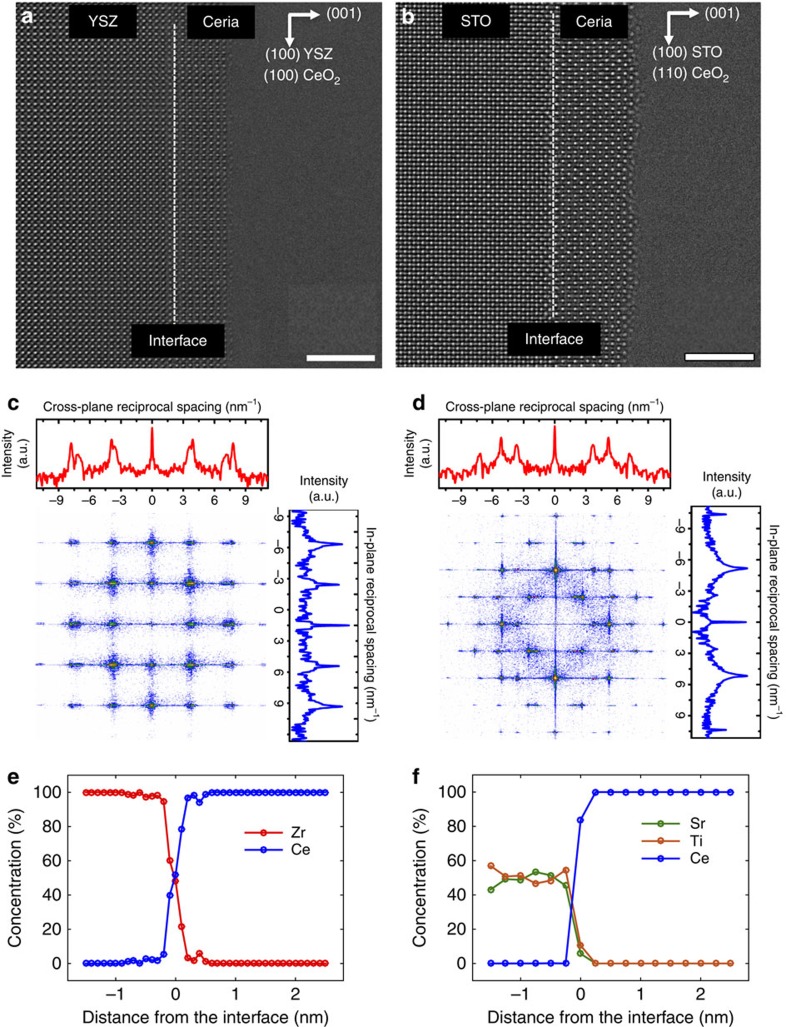
HR-TEM characterization of the CeO_2-δ_/substrate interfaces. HR-TEM images of the (**a**) CeO_2-δ_/YSZ and (**b**) CeO_2-δ_/STO interface. Bright and dark spots correspond to cations and anions, respectively. Scale bars, 3 nm. No misfit dislocations were found at either interface, confirming that the coherently strained films of ceria were stable on both YSZ and STO even after several hours of exposure to temperatures up to 550 °C and reducing oxygen partial pressures. (**c**,**d**) The FFT patterns of CeO_2-δ_/YSZ and CeO_2-δ_/STO obtained from the corresponding TEM images. Line cuts along the in-plane direction (blue curve) show singlet peaks for both films, further confirming coherency with the substrate. Along the cross-plane direction (red curve), doublet peaks are observed, due to Poisson relaxation. Energy-dispersive spectroscopy line scan obtained in scanning TEM mode across the (**e**) CeO_2-δ_/YSZ interface and (**f**) CeO_2-δ_/STO interface of the strained, ultrathin films show no signs of significant interdiffusion of cations even after APXPS measurements at elevated temperatures. Lines are for guiding the eye.

Additionally, we used high-resolution transmission electron microscopy (HR-TEM) to directly visualize the atomic structure of the interface. Cross-section TEM specimens were carefully prepared following conventional procedures (see Methods) and negative spherical aberration imaging was performed. Under these conditions, the cation columns appear brighter than the oxygen ion columns. Figure 2a,b shows typical images revealing the epitaxy of fluorite CeO_2-δ_ on YSZ in the [010] orientation, and [110]-oriented CeO_2-δ_ on [010]-oriented perovskite structure STO. The continuity of the crystal planes from substrate to the thin film is evident in both cases: there are no terminating lattice planes and hence no interfacial misfit dislocations. This was confirmed at 10 different locations along the interfaces. Moreover, in contrast with a recent theoretical calculation^39^, no secondary crystallographic phase was visible in HR-TEM images between the ultrathin ceria film and the substrates.

To quantify the degree of coherent in-plane strain and cross-plane relaxation, the fast Fourier transform (FFT) of the HR-TEM images (containing both the film and the substrate) was used (Fig. 2c,d). This is equivalent to the diffraction pattern from the periodic images. Much lower beam divergence is possible with this method than in direct nanoscale electron diffraction patterns from the specimen in the TEM. For CeO_2-δ_/YSZ, the line-cut of the FFT along [010] (in-plane direction, blue curve) shows singlet diffraction spots with a *d-*spacing of 2.55 Å (that is, 1/2 unit cell YSZ), confirming that the substrate and film have identical in-plane lattice parameter. In the line-cut along [001] (cross-plane direction, red curve), we observe doublets with *d*-spacings of 2.55 Å (1/2 unit cell YSZ) and 2.82 Å (1/2 unit cell ceria). From these data and equilibrium lattice constants, the in-plane compression and the cross-plane Poisson tension for the ceria films grown on YSZ were calculated to be 5.6% and 4.2%, respectively. This corresponds to a volume decrease of 7.1% from that of the relaxed structure. The 3.2 nm thin-film thickness is significantly greater than the expected critical thickness of 0.8 nm predicted by equilibrium theory (see Supplementary Note 1). We propose that the large nucleation barrier for misfit dislocations stabilizes the coherent film^43^.

The FFT of the HR-TEM image of CeO_2-δ_/STO is shown in Fig. 2d. The (00*l*) peaks of CeO_2-δ_ and STO are clearly separated in the diffraction pattern, and the difference in *d*-spacing is verified using a cross-plane line cut (red curve). Along the in-plane direction (blue curve), the [*hh*0] of ceria align with the 2[*h*00] reflections of STO, confirming that the film is lattice matched with the substrate in-plane. The in-plane tension and the corresponding cross-plane Poisson compression are calculated to be 2.1% and 1.3%, respectively, implying a volume increase of 2.9% over the unstrained structure. Table 1 summarizes the strain state of the ultrathin and relaxed films. The magnitude of in-plane and cross-plane elastic strains, ranging from −5.6 to 4.2%, is exceptional. The residual strain due to differential thermal expansion is expected to be 0.1%, given the film's growth temperature at 550 °C in 0.5 mTorr O_2_, since the coefficient of thermal expansion mismatch between bulk ceria and YSZ is only ∼2 × 10^−6^ K^−1^ (refs 44, 45). Thus, the measured room temperature strain state is expected to be accurate at the APXPS measurement conditions as well.

The surface topography of the strained films was examined by atomic force microscopy before and after APXPS measurements (Supplementary Fig. 1). In both films, an atomically flat terrace-and-step structure is observed. The step heights are of the order of 2.5 and 4 Å for CeO_2-δ_ films grown on YSZ and STO, respectively. The fact that the ceria films conform to the substrate terraces shows that they retain their single termination, within the detection limit of atomic force microscopy. The topography was almost identical even after sustained exposure to reducing atmospheres (H_2_/H_2_O) and temperatures up to 550 °C, suggesting that strain relief through surface roughening was not significant during the course of the experiment.

### Quantification of surface nonstoichiometry by APXPS

**Figure 3:**
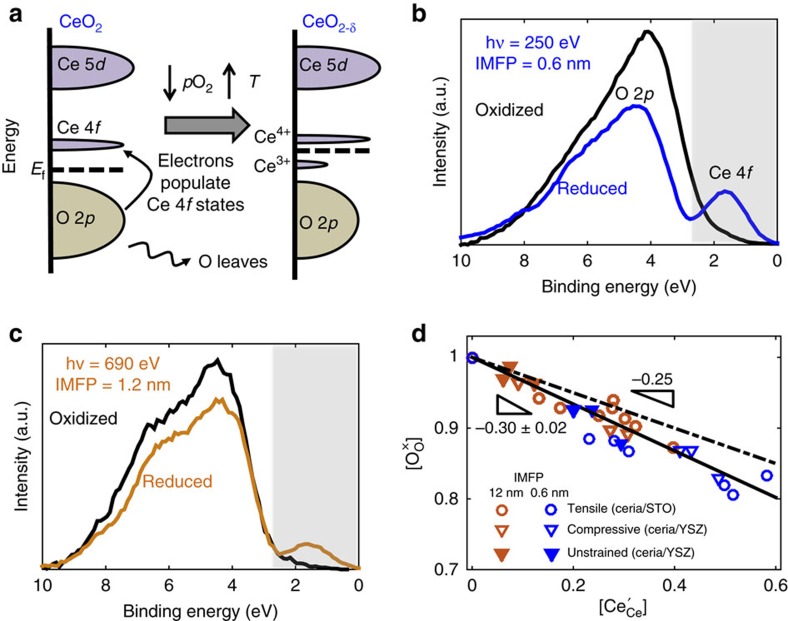
Valence-band spectra showing formation of Ce^3+^ and oxygen nonstoichiometry upon reduction. (**a**) Schematic of electronic structure changes with oxygen nonstoichiometry in CeO_2-δ_. Upon reduction, the density of O 2*p* states decreases, and that of occupied Ce 4*f* states increase proportionally. (**b**) Valence-band spectra (250 eV photons, inelastic mean free path (IMFP)=0.6 nm) at 450 °C and relatively oxidizing *p*O_2_=1.3 × 10^−4^ atm (black curve) and reducing *p*O_2_=1 × 10^−28^ atm (blue curve) showing the absence and presence of Ce 4*f* states, respectively. The spectra have been normalized by the integrated intensity of the corresponding Ce 4*d* core-level spectra. (**c**) Valence band spectra collected using 690 eV photons to probe the near-surface region (IMFP=1.2 nm) reveal the same qualitative behaviour. (**d**) A semiquantitative measure of the oxygen content, obtained from an appropriate normalization of the valence-band spectra (see Supplementary Note 2) shows an inverse correlation with Ce^3+^ concentration suggesting that oxygen vacancies form alongside Ce^3+^ at the surface and near-surface regions. Triangles and circles denote films grown on YSZ and STO, respectively. The error in 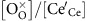, that is, the fitted slope, is estimated by combining the fitting error (95% confidence interval) as well as the uncertainties associated with converting Ce 4*f* intensity to Ce oxidation state.

The primary effect of strain is to modify the interatomic distances in a lattice that consequently modulates the potential energy landscape for electrons, and their energy levels. The tetragonal distortion, c/a of 1.1 for CeO_2-δ_/YSZ and 0.96 CeO_2-δ_/STO breaks the cubic symmetry and likely causes significant changes in the electronic structure. In CeO_2-δ_, the electronic structure has a direct impact on the redox capacity, since the formation of oxygen vacancies, the primary ionic defects, is accompanied by electron redistribution to render the system charge neutral (formation of Schottky pairs is unlikely under our experimental conditions). Specifically, the formation of an oxygen vacancy is accompanied by electron localization in the unoccupied 4*f* orbitals of two Ce^4+^. Using Kroger–Vink notation, the point defect reaction is given by:


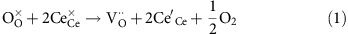


where the species, from left to right, denote O^2−^, Ce^4+^, oxygen vacancies, Ce^3+^ and gaseous oxygen, respectively. Electron correlation causes the occupied (Ce^3+^) and unoccupied (Ce^4+^) Ce 4*f* states to split (Fig. 3a). The occupied Ce 4*f* state, which can be readily measured by XPS, provides a direct measure of reducibility of ceria.

The *in situ* APXPS measurements were performed at 450 and 550 °C in both O_2_ and H_2_/H_2_O atmospheres to investigate the effect of strain on the equilibrium surface redox behaviour. 250 and 690 eV kinetic energy photoelectrons, with inelastic mean free paths of 0.6 and 1.2 nm respectively, were analysed for quantifying the extent of reduction at different depths from the surface (Supplementary Note 7). The photon energies were adjusted for the binding energies of the different core levels and valence band. Figure 3b,c shows representative valence-band spectra for the 3.2 nm CeO_2-δ_/YSZ. The spectra have been normalized by the integrated intensity of Ce 4*d* core-level spectra collected at identical kinetic energies. The reference spectra of oxidized CeO_2_ (black curve) was collected at *T*=450 °C and *p*O_2_=1.3 × 10^−4^ atm; the Ce 4*f* feature is absent. The broad feature, between 2 and 8 eV, is that of hybridized O 2*p* states. Upon lowering the oxygen partial pressure to *p*O_2_=4 × 10^−30^ atm by introducing H_2_/H_2_O, a clear Ce 4*f* peak emerges, reflecting Ce reduction^46^,^47^. Furthermore, this is accompanied by a corresponding decrease in the O 2*p* (and O 1s) intensity, a measure of the oxygen content (see Supplementary Note 2). Analysis of the unstrained and tensile samples yielded the same result, suggesting that the redox process in all films involves release of oxygen along with formation of Ce^3+^, consistent with our recent findings^48^. The near-surface fraction of Ce^3+^ was quantified using Ce 4*f* feature in the valence-band spectra normalized by the Ce 4*d* peak area, based on a previously validated procedure (see Supplementary Note 3)^46^,^47^. The fraction of oxygen sites occupied 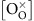 is determined by normalizing the O 1s or O 2*p* intensity against that under oxidizing conditions. Figure 3d plots semiquantitative 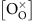 versus 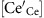 and reveals that the values for all strain states and probing depths essentially fall on the same line (values tabulated in Supplementary Table 1). The slope, a measure of the electroneutrality of the thin films within the XPS probing depth, is 0.30±0.02, close to the electroneutral site fraction of 0.25. We will return to this point later.

**Figure 4:**
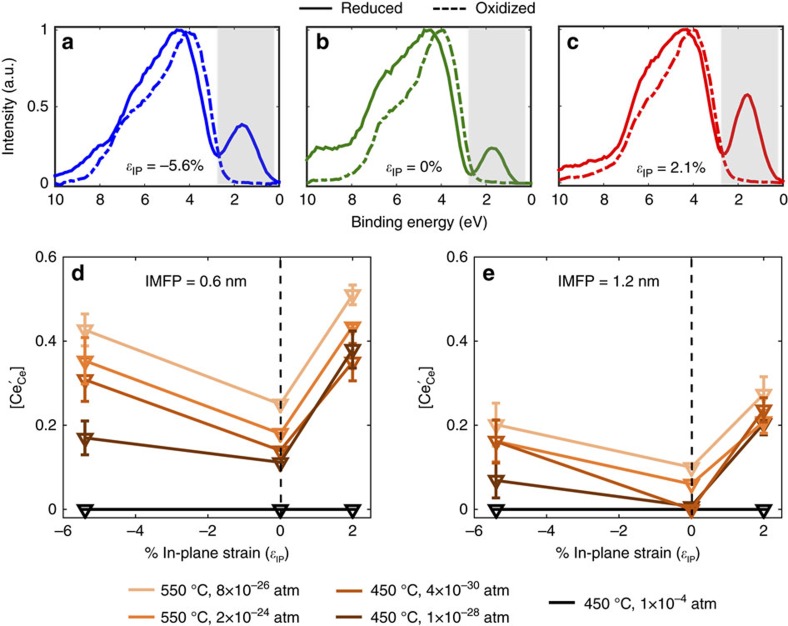
Comparison of Ce^3+^ concentration of strained and unstrained ceria films measured *in-situ*. Valence-band spectra using 250 eV photons of (**a**) compressively strained, (**b**) unstrained and (**c**) tensile strained ceria films at 490 °C. All samples are oxidized at *p*O_2_=1 × 10^−4^ atm (reference, dashed line). Upon reducing *p*O_2_ to 1 × 10^−30^ atm, the compressively strained and tensile strained films show a larger Ce 4*f* intensity compared with the unstrained film. The spectra have been normalized by the maximum of the O 2*p* feature to facilitate a direct comparison of the Ce 4*f* area. (**d**,**e**) Ce^3+^ concentration versus in-plane strain at information depths of 0.6 nm (photon energy=250 eV) and 1.2 nm (photon energy=690 eV), respectively. Error bars were obtained by repeating the experiments using films of different thicknesses. Lines are for guiding the eye.

A comparison of the valence-band spectra of the compressive, unstrained and tensile strained films, at an information depth of 0.6 nm, is shown in Fig. 4a–c. For ease of comparison, the spectra have been normalized by the maximum intensity of the O 2*p* feature. Regardless of the strain state, the ceria films are all oxidized at *T*=450 °C and *p*O_2_=1.3 × 10^−4^ atm (dashed line). Upon lowering the *p*O_2_ to 4 × 10^−30^ atm, it is apparent even without quantification that the Ce 4*f* intensity in the valence-band spectra (solid line) of the strained ceria films is substantially larger (suggesting higher reducibility) as compared with the unstrained film. Additional spectra collected at information depths of 1.2 and 1.8 nm reveal similar strain-induced enhancement. This redox behaviour was also reversible (Supplementary Fig. 2).

Figure 4d,e shows [Ce′_Ce_] as a function of in-plane strain for 0.6 and 1.2 nm information depths, respectively. At *p*O_2_=1.3 × 10^−4^ atm (black curve), [Ce′_Ce_]=0 regardless of the strain state. With decreasing oxygen chemical potential (lighter shades of brown), [Ce′_Ce_] increases. Going from 0.6 to 1.2 nm depth, [Ce′_Ce_] drops by a factor of two in strained ceria, and even more so in unstrained ceria. At both information depths, the dependence of [Ce′_Ce_] on strain is consistently non-monotonic. The effect of biaxial tension is nearly twice as potent as that of compression, judging by the slope of the ‘V' curves. In other words, 2.1% tension and 5.6% compression lead to similar increase in [Ce′_Ce_] relative to the unstrained value.

At first glance, the enhancement in reducibility with respect to the unstrained sample seems small, since the reported increases in conductivity or surface reactivity often deemed significant are well over an order of magnitude. However, we note that the surface of ceria, even in the absence of strain, is already enriched in Ce^3+^ compared with the bulk^46^,^47^. Straining the lattice leads to further enrichment of surface oxygen vacancies and Ce^3+^. The fourfold increase in the surface [Ce′_Ce_] of ceria films upon biaxial straining translates to a decrease in the oxygen vacancy formation energy by ∼0.4 eV per O (see Supplementary Note 4). For Sm-doped CeO_2-δ_, Chueh *et al*.^47^ determined the reduction enthalpy at the surface to be 2.9 eV, nearly 1 eV lower than that in the bulk (>4 eV). Both compressive and tensile strains lead to a further decrease by 15%. A similar non-monotonic reduction in vacancy formation energies has been reported computationally by Donner *et al*.^40^ in La_0.5_Sr_0.5_CoO_3–δ_ under compression and tension.

### Decoupling strain and space-charge effect on defect concentration

**Figure 5:**
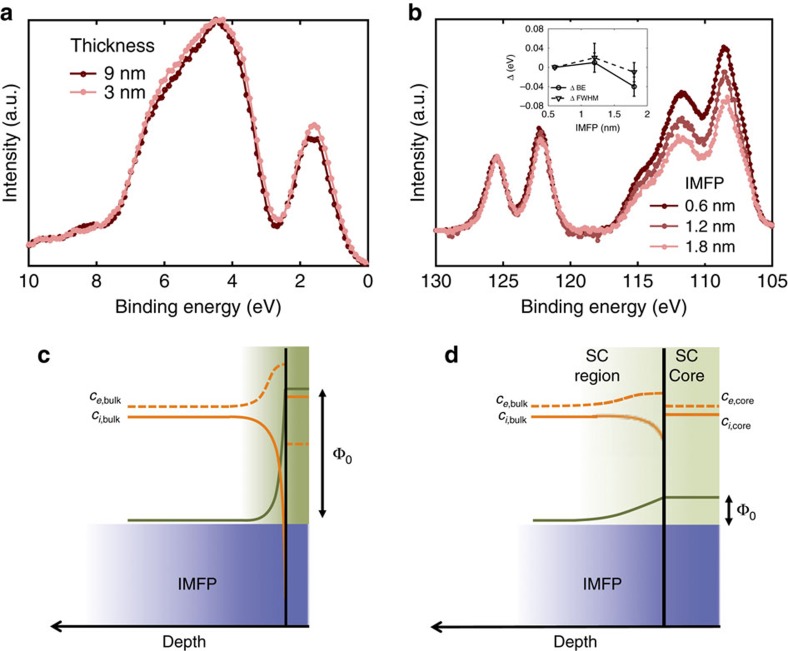
Probing the effect of the film/substrate interface on the reducibility of the ceria films. (**a**) Comparison of the valence-band spectra of 3 and 9 nm ceria films on STO. Information depth=0.6 nm, *T*=550 °C, *p*O_2_=8 × 10^−26^ atm. The spectra are quantitatively identical, suggesting lack of substrate induced effects on the measured oxygen nonstoichiometry. (**b**) Ce 4*d* spectra for the 3 nm CeO_2-δ_/STO film at information depths of 0.6 nm (hν=370 eV), 1.2 nm (hν=790 eV) and 1.8 nm (hν=990 eV) under identical conditions as in **a**. The spectra are normalized by the intensity of the highest binding energy peak (126 eV), such that the lower binding energy peaks are an indication of relative concentration of Ce^3+^. Inset shows binding energy shift of the peaks at ∼122 and ∼126 eV across the three probing depths, and the change in FWHM. Errors bars were obtained by averaging over two peaks and two samples. (**c**,**d**) Schematics illustrating the likely space-charge profiles in the information depth probed by APXPS, consistent with our experiments and simulations. Symbols are explained in the Supplementary Information. (**c**) XPS probes a SC layer that is significantly thinner than the inelastic mean free path (IMFP) of 0.6 and 1.2 nm, rendering the defect chemistry in the probing depth charge neutral. (**d**) XPS mainly probes the SC core (and a part of the SC layer) with a very weak SC potential such that deviation from charge neutrality is small.

The surprising nature of these findings on ultrathin films begs the question of whether the enhancement in redox capacity is dominated by electrostatic or chemical effects arising from the ceria/substrate or ceria/gas interface. Next, we detail several experimental observations and simulations indicating that this is not likely the case. We first compare the redox behaviour of 3.0 nm versus 9.0 nm ceria on STO (Supplementary Fig. 3), both of which are under identical biaxial tension. Figure 5a shows the valence-band spectra at 550 °C and *p*O_2_=2 × 10^−24^ atm. The variation of [Ce′_Ce_] with strain and chemical potential are quantitatively unaffected by the film thickness. We also compared the redox behaviour of ∼5 nm ceria grown on (001) La_0.18_Sr_0.82_Al_0.59_Ta_0.41_O_3_ (LSAT, 0.7% biaxial tension) and 350 nm ceria grown on YSZ (the unstrained reference). APXPS of ultrathin CeO_2-δ_/LSAT revealed nearly identical valence-band spectra and [Ce′_Ce_] as the 350 nm CeO_2-δ_/YSZ over a range of reducing atmospheres (Supplementary Fig. 4 and Supplementary Note 5). Hence, the nature of the substrate and the proximity of the buried interface do not have a discernible impact on the measured redox capacity.

To further confirm that the ceria/substrate interface does not contribute to the strain-dependent redox capacity, we varied the probing depth by changing the photoelectron kinetic energy during APXPS. Figure 5a shows the Ce 4*d* spectra for 3 nm CeO_2-δ_/STO at inelastic mean free paths of 0.6, 1.2 and 1.8 nm, respectively, normalized by the two highest binding energy peaks (that do not have any contribution from Ce^3+^). At greater information depths, the intensity of the lower binding energy peaks, which scale with total Ce content, decrease. This suggests that the extent of reduction is greatest at the surface and decreases with probing depth, providing further evidence that the influence of the buried interface on the enhanced reducibility, beyond introduction of uniform elastic strain, is negligible.

For completeness, we also examine the possibility that cations could diffuse from the substrate to the ceria films during growth and/or characterization at elevated temperatures. Diffusion of Zr or Ti into the ceria film, for example, could lead to the formation of Zr or Ti substituted ceria, both of which have a markedly lower enthalpy of reduction, and would hence favour higher [Ce′_Ce_]^49^,^50^. Using scanning TEM (STEM) analysis with energy-dispersive spectroscopy (with a detection limit of ∼1 at.%), we find no trace of Zr, Sr or Ti in the ceria films even after APXPS measurements at elevated temperatures beyond 0.3 nm from the interface (Fig. 2e,f). For a 1% Zr or Ti interdiffusion into ceria, bulk thermodynamics^49^ suggests that the Gibbs free energy of reduction will change by less than 1–2% depending on the oxygen nonstoichiometry.

Finally, we consider the possibility that strain is coupled to space-charge effects at the surface of the film. An electrostatic potential gradient in the near-surface region modulates the binding energy of photoelectrons as a function of depth. The effective photoelectron spectrum, which exponentially integrates signal from different depths in the space-charge region, exhibits a broadening in the full-width at half maximum (FWHM) and a shift in the binding energy (Shavorskiy *et al*., in preparation). Figure 5b overlays Ce 4*d* core-level spectra collected at information depths of 0.6, 1.2 and 1.8 nm, respectively, at 550 °C for 9 nm CeO_2-δ_/STO. The shift in binding energies of the spectra relative to one another (<0.04 eV) and the change in FWHM (<0.03 eV) with probing depth are within the error of APXPS measurement.

For a more quantitative assessment, we simulated the oxygen vacancy and electron distribution in the near-surface region using a thermodynamically consistent space-charge (SC) model (see Supplementary Fig. 5 and Supplementary Note 6), and calculated the corresponding X-ray photoelectron spectra. Taking a range of electron and oxygen vacancy segregation energies between 0 and −1.5 eV as the driving force for SC formation, we mapped out the resulting electrostatic potential in the SC region and in the core, and the concentration profiles (Supplementary Figs 6 and 7). We simulate the [V_o_^˙̇^]/[Ce′_Ce_] probed by XPS (Fig. 3d), which shows an experimental value of 0.30±0.02, in slight excess of the expected 0.25 in the electroneutral limit. More importantly, the [V_o_^˙̇^]/[Ce′_Ce_] ratio is close to 0.30±0.02 irrespective of the biaxial strain state of the oxide films, and the XPS probing depth (0.6 versus 1.2 nm). Based on the simulations, this experimental result corresponds to a negligible space-charge potential (*Φ*_0_) of < 0.1 eV (in the limit that XPS only probes the SC core) or a *Φ*_0_ of ∼0.2 eV and SC width of 0.2 nm (in the limit that XPS only probes the SC region/bulk). From this comparison, we conclude that (1) if the XPS probes the SC region, it is likely too thin to contribute significantly to the observed strain effects, and that the observed behaviour correspond primarily to defects in the bulk (Fig. 5c); (2) or, if XPS probes mostly the surface core, the experimental [V_o_^˙̇^]/[Ce′_Ce_] ratio suggests a very weak *Φ*_0_ (Fig. 5d). Moreover, the fact that the experimental [V_o_^˙̇^]/[Ce′_Ce_] ratio does not depend on misfit strain confirms that SC effect does not explain the unusual strain dependence of the defect concentrations that we observed.

We note that having thin films with uniform strain that is independent of thickness is key towards understanding the role of the interface on the redox capacity. Finally, we note that *ex situ* observation of CeO_2-δ_/YSZ interface being reduced^38^ is not inconsistent with our interpretation, which is that biaxial strain interacts with the rest of the film more strongly. Having ruled out the effect of the buried interface, next we focus on clarifying the role of biaxial strain.

### *Ab initio* vacancy thermodynamics under biaxial strain

**Figure 6:**
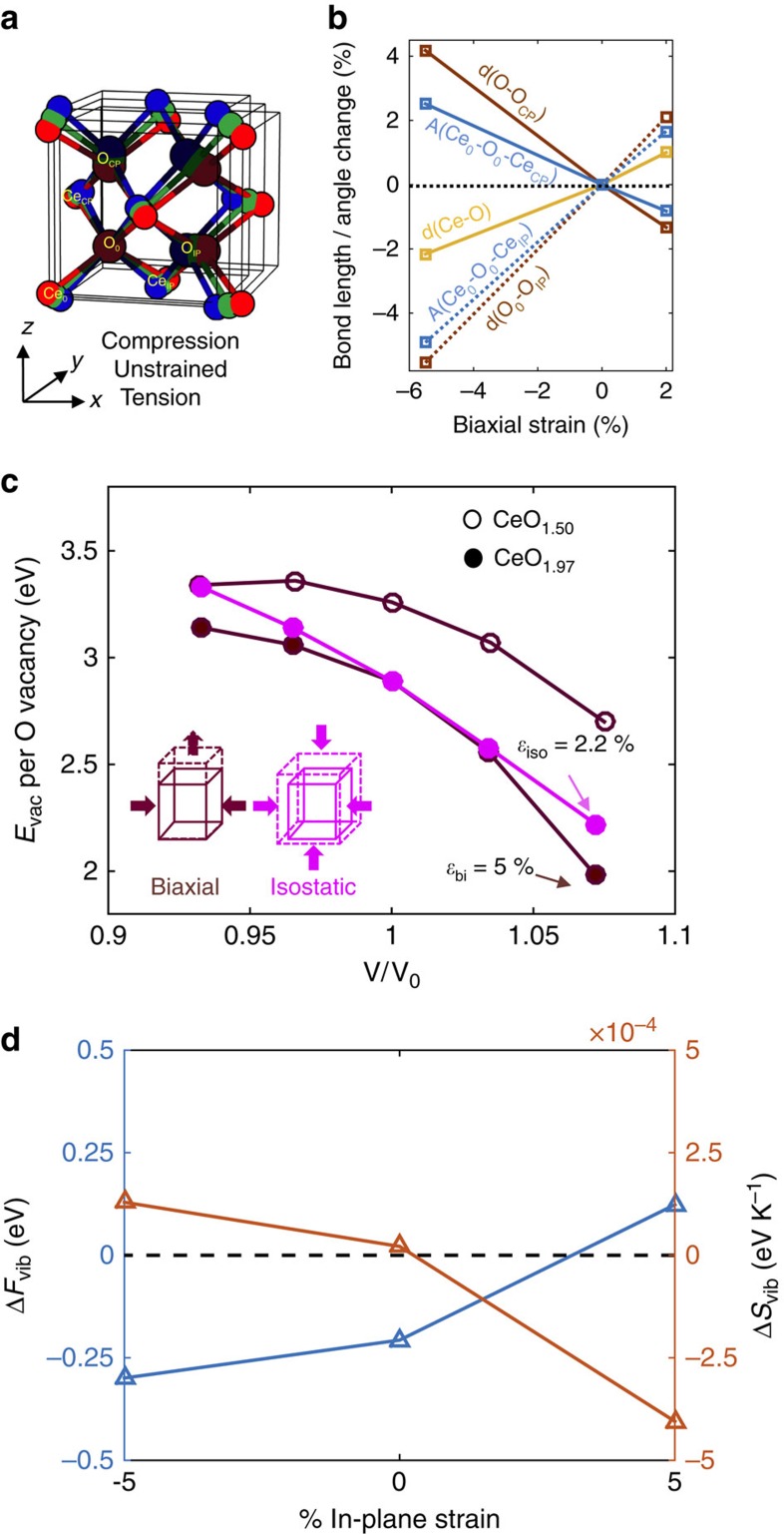
Role of biaxial strain-induced tetragonal distortion. (**a**) Schematic of the unit cell of ceria in the absence of strain (green), under compression (blue) and tension (red). Only the front half is shown for clarity. (**b**) Change in Ce-O, O_0_-O_IP_ and O_0_-O_CP_ distances (denoted ‘d') and Ce_0_-O_0_-Ce_IP_ and Ce_0_-O_0_-Ce_CP_ bond angles (denoted ‘A') as a function of biaxial strain. While the change in Ce-O distance is monotonic with biaxial strain and does not split, the other quantities split into doublets whose values are distributed on either side of the unstrained values. Lines are for guiding the eye. (**c**) Vacancy formation energy (*E*_vac_) per oxygen released in bulk ceria as a function of relative volume (with respect to the unstrained bulk) under biaxial and isostatic strain for CeO_1.97_ (1 oxygen vacancy in a 2 × 2** × **2 supercell) and CeO_1.50_ (2 oxygen vacancies in a unit cell). (**d**) Vibrational free energy and entropy change per oxygen released in bulk ceria calculated under the harmonic approximation at 550 °C for three different strain states. In all figures, lines are for guiding the eye.

The most direct impact of biaxial strain on a cubic crystal is to introduce tetragonal distortion, shifting the electronic energy levels and lifting band degeneracy. Ligand field splitting, which leads to a pronounced rearrangement of electronic energies in 3*d* transition metal perovskites, is negligible in ceria, as evidenced by the narrow Ce 4*f* bandwidth in *ab initio* calculations^51^. Under biaxial strain, the bond lengths and bond angles within the oxygen-centred tetrahedra and the spacing between them are modified. Figure 6a illustrates the three stoichiometric unit cells under compression (blue), no strain (green) and tension (red). Also plotted alongside in Fig. 6b are percentage changes in the Ce-O and O-O distances, Ce-O-Ce angles and the spacing between tetrahedra as a function of strain, using the average crystal structure obtained from TEM. For reference, the oxygen at (0.25, 0.25, 0.25) position in unit cell coordinates has been set as the origin in all of the structures. Three salient features emerge. First, the Ce-O distances vary monotonically with biaxial strain and remain degenerate, by virtue of being oriented along the [111] direction. Second, the tetrahedron distorts such that distinct in-plane (Ce_0_-O_0_-Ce_IP_) and cross-plane (Ce_0_-O_0_-Ce_CP_) angles emerge, where subscripts ‘IP', ‘CP' and ‘0' denote in-plane, cross-plane, and reference species, respectively. While the cross-plane bond angle is greater than the regular tetrahedral angle of 109.47° under compression, the in-plane angle is larger under tension. Third, the O-O distance, that is, the spacing between tetrahedra, is also split into in-plane and cross-plane values that scale oppositely with strain. Based on these simple geometric observations, two distinct oxygen vacancy pairs can be identified for nonstoichiometric ceria, whose formation energetics likely exhibit different dependencies on strain. Biaxial strain also likely exacerbates the tendency for oxygen vacancies to form along certain directions.

Yet another symmetry consideration ties into the relative stabilities of the two-oxidation states of Ce. The 4+ oxidation state is favoured in the eightfold coordinated cubic fluorite structure, while the 3+ oxidation state is more stable in the sixfold coordinated, hexagonal sesquioxide structure. Oxygen nonstoichiometry provides a natural means to lower the oxidation state of Ce to 3+ to better accommodate the lower symmetry under biaxial strain, both compressive and tensile. The competing effects of chemical expansion and symmetry constrained Ce^3+^ formation could likely lead to the shallower dependence of [Ce′_Ce_] on compressive strain.

To explore the role of tetragonal distortion under large biaxial strain, we performed DFT plus Hubbard *U* (DFT+*U*) calculations of oxygen vacancy formation energies (*E*_vac_) in the relatively simpler bulk model system. Specifically, we subject bulk CeO_2-δ_ to biaxial versus isostatic strain (see Methods for details). At each value of biaxial strain, Poisson relaxation and chemical expansion were explicitly accounted for. The magnitude of the isostatic strain was chosen to match the optimized cell volume under biaxial strain for the stoichiometric and nonstoichiometric structures, enabling a meaningful comparison between the two. For instance, 5.0% biaxial compression, after accounting for Poisson relaxation, corresponds to an equal change in volume as 2.2% isostatic compression. Figure 6c illustrates *E*_vac_ as a function of volume (normalized by the corresponding unstrained value), and the values are also tabulated in Supplementary Table 2. Even for the bulk, the difference between isostatic versus biaxial—a direct consequence of tetragonal distortion—is striking beyond a volume change of 2 to 3%. For small volume changes, the two modes of straining the lattice result in a monotonic, almost-linear decrease in oxygen vacancy formation energy, in agreement with a recent study on uniaxially strained bulk ceria by Sun *et al*.^30^ Under large biaxial strain, on the other hand, the behaviour is asymmetric under compression and tension. Specifically, the oxygen vacancy formation energy displays a significantly shallower dependence on compression compared with tension. Interestingly, the vacancy formation energies approach an inflection point around a compressive strain of ∼5% (similar to the coherent CeO_2-δ_/YSZ). We also computed the vacancy formation energies at the (100) terminated surface of ceria under biaxial strain (see Methods for details of the surface calculations). The most stable surface vacancies (of the many distinct positions), summarized in Supplementary Table 3, have lower formation energies relative to the bulk, in agreement with the literature, but the dependence on biaxial strain is similar to that of the bulk.

To probe the interplay between biaxial strain and oxygen vacancy–vacancy interaction, we computed the formation energy of a second oxygen vacancy in a unit cell of bulk ceria (that is, δ=0.50). We are aware that disordered ceria is likely not stable in its cubic/tetragonal crystal structure at such high oxygen nonstoichiometry. Nevertheless, to achieve a large vacancy concentration while keeping the electron localization problem tractable, this calculation remains insightful. The nonlinearity in the dependence of oxygen vacancy formation energies on volume persists under large biaxial strain (Fig. 6c). Moreover, a weakly non-monotonic behaviour becomes apparent at δ=0.50 compared with δ=0.03 under large biaxial compression. DFT+*U* calculations reveal that for all values of biaxial compressive strain, oxygen vacancy ordering is favoured in the cross-plane direction to minimize vacancy–vacancy repulsion. For low values of biaxial tension (up to 2.5%), orienting oxygen vacancies in the cross-plane direction is still favoured, as the chemical expansion due to the significantly larger Ce^3+^ ions is better accommodated along the unconstrained cross-plane direction. For large biaxial tensile strain, there is no apparent preference in vacancy ordering in the in-plane and out-of-plane directions, as expected. The coupling between biaxial strain and vacancy ordering/association can be inferred from Fig. 6c; cross-plane ordered vacancies become relatively more favoured under large biaxial compression compared with tension. This suggests that defect association, enabled by high vacancy content, could be serving as a strain relaxation mechanism^34^,^35^.

Chemical expansion associated with oxygen vacancy formation makes the process vibrationally stabilized in the absence of geometric constraints^52^,^53^. To investigate the effect under biaxial strain, we computed the vibrational entropy and free energy changes with oxygen vacancy formation as a function of temperature under the harmonic approximation at −5, 0 and 5% biaxial strain. Figure 6d depicts the vibrational free energy and entropy change per oxygen as a function of biaxial strain at 550 °C (the corresponding harmonic phonon density of states for the three strain states and two stoichiometries are plotted in Supplementary Fig. 11). The difference in the oxygen vibrational free energy (*F*_vib_) between compressively strained and unstrained ceria, ∼0.1 eV, is comparable in magnitude to the difference in the enthalpy (*E*_vac_), also ∼0.1 eV; between tensile strained and unstrained ceria, the difference in the *F*_vib_, ∼0.3 eV, is likewise significant in magnitude compared with the difference in the *E*_vac_, ∼0.6 eV. However, in contrast to *E*_vac_, *F*_vib_ becomes more favourable as lattice constants are decreased. Given the relative importance of vibrational contribution with increasing temperature, our calculations suggest that this could be another important factor in determining how compression enhances the redox capacity relative to the unstrained ceria.

With these insights, we can now look at the effect of biaxial strain on the redox properties as being multipronged, affecting energetics, vibrations and ordering of oxygen vacancies. Under biaxial tension, the larger lattice volume and the lowered crystal symmetry inherently favour reduction of ceria. Under biaxial compression, Ce^3+^ formation is hindered if the strain coupling with volume is the only driving force. However, the ability of 3+ oxidation state of Ce to better accommodate the tetragonal distortion from biaxial strain and the lowering of vibrational free energy favour the creation of oxygen vacancies. The finding that the enhanced reducibility is insensitive to the type of biaxial strain could explain the observation of Song *et al*.^38^ about the reduction of Ce in the heterogeneous strain fields surrounding a misfit dislocation.

To conclude, we fabricated ultrathin CeO_2-δ_ films that are coherently strained under large biaxial compression on (001) YSZ (−5.6%) and tension on (001) STO (2.1%), and characterized their oxygen redox chemistry. The *in situ* APXPS at 450–550 °C and conditions relevant for water splitting and hydrogen oxidation reactions revealed a dramatic, fourfold enhancement in the surface redox capacity of the ceria films under both compression and tension. Geometrical considerations and *ab initio* calculations indicate that biaxial strain affects the redox capacity not only by its coupling with chemical expansion, but also by breaking the cubic symmetry of ceria through tetragonal distortion. This non-monotonic enhancement in the oxygen nonstoichiometry could be useful in applications relying on redox capacity. The decrease in oxygen vacancy formation energy by ∼0.4 eV observed in this study could lower the operating temperature and/or overpotential of catalysts and electrocatalysts significantly. Our insights into the role of strain may guide the rational redox engineering using nanoscale strain fields such as that found near dislocations and in multilayer structures of nonstoichiometric oxides. In particular, the strain-enhanced redox capacity being largely agnostic to the type of strain could relax material requirements for fabrication.

## Methods

### Materials and synthesis

High-quality, ultrathin ceria films were grown on atomically flat single crystal (001) Y_0.19_Zr_0.81_O_1.905_ (YSZ, CrysTec), and (001) SrTiO_3_ (STO, Shinkosa) substrates via pulsed-laser deposition (PLD/MBE 2300, PVD products). While ceria and YSZ crystallize in the cubic fluorite structure under conditions relevant in this study, STO adopts a cubic perovskite structure. The lattice constants of ceria, YSZ and STO are 5.414, 5.142 and 3.905 Å, respectively. The PLD target was pressed uniaxially from commercial CeO_2_ powder (Sigma Aldrich, 99.995%) and sintered at 1350 °C in 21% O_2_ balance Ar for 5 h. Single-terminated terraces on YSZ and STO surfaces were obtained using the following annealing procedures. For YSZ, the as-received substrates were sonicated in isopropanol for 15 min, followed by annealing in ultra-high purity Ar at 1,250 °C for 12 h. The STO substrates were sequentially sonicated in isopropanol for 15 min and 18 MΩ water for 30 min. Subsequently, they were etched in 1:6 HF/NH_4_F mixture, and annealed at 1,000 °C, in Ar atmosphere for 2 h to obtain atomically flat terraces.

Subsequently, ceria films were grown in 0.5 mTorr O_2_ at ∼500 °C in a PLD chamber equipped with a KrF excimer laser. Then, 2 J cm^−2^ laser fluence, 1 Hz repetition rate (10 Hz for the bulk-like films) and 100 mm target–substrate distance were used. After the deposition, the samples were cooled to room temperature in 0.5 mTorr O_2_. The unstrained ceria film (350 nm) was grown on an as-received (001) YSZ substrate.

### X-ray diffraction

In accordance with the substrate orientation, cross-plane X-ray diffraction scans (Xpert Pro, PANalytical, Cu Kα) revealed only (002) family of peaks for all the ceria films, Supplementary Fig. 8. Phi scans showed that CeO_2_ grows epitaxially with perfect alignment registry on YSZ, and with a 45° in-plane rotation on STO ([100]_ceria_||[110]_STO_). Rocking curve scans revealed a FWHM of 0.4° for the bulk-like films and 0.02° for the ultrathin films. The thickness of the ultrathin films was obtained using X-ray reflectivity, and independently verified using Scherrer equation on the peak broadening of the (002) reflection. The thickness of the bulk-like film was measured to be 350 nm using stylus profilometry. Non-contact atomic force microscopy (XE-100, Park) was performed to characterize the topography of the annealed substrates before and after deposition, and after APXPS.

### Transmission electron microscopy

The cross-section specimen was glued and mechanically polished to 15 μm thickness and ion-milled to electron transparency using a Gatan PIPS II System. A 5 keV argon ion beam was used to create a hole in the center of the specimen with an incidence angle of 5°. Then, the Ar ion energy was gradually reduced to 0.1 keV to remove the surface amorphous layer. TEM experiments were performed using a Titan 80-300 environmental transmission electron microscope (FEI) operated at 300 kV. The instrument is equipped with a spherical aberration corrector in the imaging (objective) lens. Aberration-corrected TEM imaging was performed using a Cs value of ∼−13 μm, and all images presented were acquired at a positive defocus condition (negative Cs imaging). STEM analysis with X-ray energy-dispersive spectroscopy was carried out by operating the microscope in scanning mode without the monochromator. The STEM probe size was ∼0.2 nm in diameter.

### Ambient pressure X-ray photoelectron spectroscopy

Synchrotron APXPS measurements were conducted at beamline 11.0.2 of the Advanced Light Source^54^, Lawrence Berkeley National Laboratory. A custom-designed sample holder equipped with a ceramic heater was used^55^. The heater current and voltage settings to maintain constant temperature under different gas environments were determined using a YSZ substrate sputter-coated with Pt and measuring its surface temperature with an optical pyrometer. To eliminate variations in gas pressure and temperature between samples, the strained and unstrained films were always loaded together and characterized in the same experiment. The samples were heated to 450 °C in 100 mTorr O_2_ to remove adventitious carbon on the surface. This condition was used to measure the reference spectra under oxidizing conditions. Ce *4*d core-level and valence-band spectra were collected *in situ* at 450 °C and 550 °C under 100 mTorr O_2_ and 55 mTorr H_2_-H_2_O atmospheres, using H_2_/H_2_O ratios of 1:10 and 1:1. The resulting *p*O_2_, ranging between 2 × 10^−24^ and 4 × 10^−30^ atm, was monitored using a vacuum compatible zirconia-based oxygen sensor (Zirox Gmbh). Photon energies were suitably chosen to probe different information depths. For surface-sensitive measurements, 250 eV kinetic energy photoelectrons were used, while for greater depths, 690 and 890 eV photoelectrons were used. The 4*f*_7/2_ peak of an Au foil in contact with the thin films and Fermi coupled to the electron analyser was used for binding energy calibration. In valence-band XPS, switching from oxidizing to reducing condition, we observed that the equilibration time of the photoelectron spectra is 600 s. YSZ is irreducible under the conditions of this work. This was also verified by APXPS. For STO, chemical inertness was verified by the lack of Ti^3+^ signal in Ti 2*p* XPS. The [Ce′_Ce_] quantification was performed using a procedure outlined in the Supplementary Note 3 and Supplementary Fig. 9.

### Computational methods

DFT calculations within the Hubbard *U* approach (DFT+*U*) were carried out using the Vienna *Ab initio* Simulation Package (VASP, version 5.3.2). The core electrons of Ce and O atoms were replaced by projector-augmented wave potentials, whereas their valence electrons (5*s*, 5*p*, 4*f* and 6*s* for Ce, and 2*s*, 2*p* for O) were expanded in plane waves with a kinetic energy cutoff of 500 eV. To correctly describe the strongly correlated character of the Ce-4*f* orbitals, the Hubbard effective term *U*_eff_(Ce)=4.5 eV was added to the Perdew–Burke–Ernzenhof exchange correlation functional through the rotationally invariant approach proposed by Dudarev *et al*.^56^. This *U*_eff_ (Ce) value was adopted from self-consistent calculations by Fabris *et al*.^51^ using the linear response approach, and has been previously shown to provide satisfactory results^57^.

The lattice parameter in the bulk fluorite structure of ceria was optimized using a Γ-centred Monkhorst-Pack *k*-point grid of size 9 × 9 × 9. The lattice parameter of *a*_calc_=5.497 Å is in good agreement with previous computational studies employing similar parameters^58^. The effect of strain on the oxygen vacancy formation energy was modelled using 2 × 2 × 2 and 1 × 1 × 1 unit cells, and sampling the Brillouin zone with a Γ-centred 3 × 3 × 3 and 9 × 9 × 9 *k*-point grids, respectively. Surface calculations were performed using a 6 unit cell thick slab of *p*(1 × 1)−CeO_2_(100) surface separated by at least 13 Å of vacuum to closely represent the experimental system. To maintain the stoichiometry of this surface and avoid the buildup of a dipole moment normal to the surface, we adopted the same strategies employed in previous theoretical studies^59^,^60^ and displaced half of an oxygen monolayer from the surface to the bottom of the slab. The Brillouin zone in this case was sampled with a Γ-centred 3 × 3 × 3 *k*-point mesh.

The effect of strain on surface oxygen vacancy formation was carried out by imposing biaxial strains of −5.0, 0.0 and +5.0%, and calculating the energy required to remove one oxygen atom. This corresponds to a vacancy concentration of 11% (1 out of 9 oxygen atoms missing) of the topmost 12 Å of the surface slab. We also explored all the possible O-vacancy positions within this probing depth (see Supplementary Fig. 10) for each strain to obtain the lowest energy configuration.

Spin-polarized calculations were performed and the supercell geometries were relaxed until a threshold force of 0.02 eV A^−1^ was fulfilled. Details of electron localization can be found in the Supplementary Note 8. The bulk structures under biaxial strain were modelled by constraining the (*x*, *y*) lattice parameters. The cross-plane relaxation (*z*) was obtained by performing multiple constant-volume calculations at different values of ‘*z*', and fitting a parabola to a plot of total energy versus ‘*z*'. The isostatic strain was chosen to match the volume of each structure obtained after energy minimization under biaxial strain.

Oxygen vacancy formation energies reported in this work were calculated as follows:


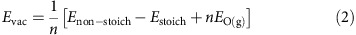


where *E*_non-stoich_ and *E*_stoich_ denote total energies of the simulated defect and defect-free cells, respectively, *n* denotes the number of oxygen vacancies and *E*_O(g)_ denotes half the energy of an isolated oxygen gas molecule. The ordering of the oxygen vacancies along the in-plane and cross-plane directions was investigated using the 1 × 1 × 1 unit cell and creating a second adjacent oxygen vacancy in both directions of the space.

Phonon calculations were performed by means of density functional perturbation theory implemented in the VASP code. With the force constants output by VASP, we obtained the dynamical matrix at different *q*-points and the phonon density of states (DOS) using Phonopy^61^. Supplementary Fig. 11 shows the phonon DOS for a unit cell of bulk ceria without and with an oxygen vacancy, at strains of −5, 0 and 5%. Upon vacancy formation, compressive strained bulk ceria exhibits the largest softening of phonon frequencies (difference between the nonstoichiometric and stoichiometric phonon DOS). Supplementary Table 4 summarizes the extent of lattice expansion accompanying vacancy formation for the three strain states. For nonstoichiometry change from 0 to 0.25, there is 4.4% expansion along the *c*-axis under 5% compression as compared with 3.7% and 3.5% under no strain and 5% tension, respectively. Using the harmonic approximation, the vibrational free energies were calculated from the phonon DOS using the following equation:


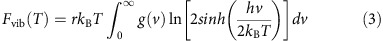


where *r* is the number of degrees of freedom, *g(ν)* is the normalized phonon density of states and *k*_B_ is the Boltzmann constant. The partial molar vibrational free energy of oxygen was computed as the difference in free energies of the nonstoichiometric and stoichiometric structures after accounting for the loss in the phonon modes due to the missing oxygen atoms. Mathematically, it can be expressed as:


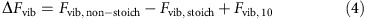


The entropy change *S*_vib_ was calculated by taking the derivative of *F*_vib_ with respect to *T*.

### Data availability

The data supporting the findings in this study are available from the corresponding author on request.

## Additional information

**How to cite this article:** Gopal, C. B. *et al*. Equilibrium oxygen storage capacity of ultrathin CeO_2-δ_ depends non-monotonically on large biaxial strain. *Nat. Commun.* **8**, 15360 doi: 10.1038/ncomms15360 (2017).

**Publisher's note:** Springer Nature remains neutral with regard to jurisdictional claims in published maps and institutional affiliations.

## Supplementary Material

Supplementary Information Supplementary Figures, Supplementary Tables, Supplementary Notes and Supplementary References

## Figures and Tables

**Table 1:** Summary of crystallographic information obtained from TEM and X-ray diffraction.

	**Substrate**	***ɛ***_**IP**_	***ɛ***_**CP**_	**Δ*****V***	**c/a**	**Thickness**
Compression	(001) YSZ	−5.6%	4.2%	−7.1%	1.1	3.2 nm
Unstrained	(001) YSZ	0%	0%	0%	0	350 nm
Tension	(001) STO	2.1%	−1.3%	2.9%	0.96	3.0 nm

*ɛ*_IP_ and *ɛ*_CP_ denote the % strain along in-plane and cross-plane directions, respectively (minus sign denotes compressive strain), Δ*V* denotes change in unit cell volume with respect to the unstrained structure and c/a denotes the tetragonal distortion (ratio of cross-plane to in-plane lattice constant).

## References

[b1] Jacobson A. J. Materials for solid oxide fuel cells. Chem. Mater. 22, 660–674 (2009).

[b2] Trovarelli A. Catalytic properties of ceria and CeO_2_-containing materials. Catal. Rev. 38, 439–520 (1996).

[b3] Scheffe J. R. & Steinfeld A. Oxygen exchange materials for solar thermochemical splitting of H_2_O and CO_2_: a review. Mater. Today 17, 341–348 (2014).

[b4] Waser R. & Aono M. Nanoionics-based resistive switching memories. Nat. Mater. 6, 833–840 (2007). 17972938 10.1038/nmat2023

[b5] Tuller H. L. & Bishop S. R. Point defects in oxides: tailoring materials through defect engineering. Annu. Rev. Mater. Res. 41, 369–398 (2011).

[b6] Li J., Shan Z. W. & Ma E. Elastic strain engineering for unprecedented materials properties. MRS Bull. 39, 108–117 (2014).

[b7] Chu M., Sun Y. K., Aghoram U. & Thompson S. E. Strain: a solution for higher carrier mobility in nanoscale MOSFETs. Annu. Rev. Mater. Res. 39, 203–229 (2009).

[b8] Bozovic I., Logvenov G., Belca I., Narimbetov B. & Sveklo I. Epitaxial strain and superconductivity in La_2-x_Sr_x_CuO_4_ thin films. Phys. Rev. Lett. 89, 107001 (2002). 12225215 10.1103/PhysRevLett.89.107001

[b9] Locquet J. P. . Doubling the critical temperature of La_1.9_Sr_0.1_CuO_4_ using epitaxial strain. Nature 394, 453–456 (1998).

[b10] Schlom D. G. . Strain tuning of ferroelectric thin films. Ann. Rev. Mater. Res. 37, 589–626 (2007).

[b11] Schlom D. G. . Elastic strain engineering of ferroic oxides. MRS Bull. 39, 118–130 (2014).

[b12] Han J. W. & Yildiz B. Mechanism for enhanced oxygen reduction kinetics at the (La,Sr)CoO_3_-delta/(La,Sr)(2)CoO_4+δ_ hetero-interface. Energ. Environ. Sci. 5, 8598–8607 (2012).

[b13] Kushima A., Yip S. & Yildiz B. Competing strain effects in reactivity of LaCoO_3_ with oxygen. Phys. Rev. B 82, 115435 (2010).

[b14] Kubicek M. . Tensile lattice strain accelerates oxygen surface exchange and diffusion in La_1-x_Sr_x_CoO_3-δ_ thin films. ACS Nano 7, 3276–3286 (2013). 23527691 10.1021/nn305987x PMC3635458

[b15] Petrie J. R. . Enhanced bifunctional oxygen catalysis in strained LaNiO3 perovskites. J. Am. Chem. Soc. 138, 2488–2491 (2016). 26866808 10.1021/jacs.5b11713

[b16] Shi Y. U., Bork A. H., Schweiger S. & Rupp J. L. M. The effect of mechanical twisting on oxygen ionic transport in solid-state energy conversion membranes. Nat. Mater. 14, 721 (2015). 26076303 10.1038/nmat4278

[b17] Kushima A. & Yildiz B. Oxygen ion diffusivity in strained yttria stabilized zirconia: where is the fastest strain? J. Mater. Chem. 20, 4809–4819 (2010).

[b18] Lee S. . Strain tuning and strong enhancement of ionic conductivity in SrZrO_3_-RE_2_O_3_ (RE=Sm, Eu, Gd, Dy, and Er) nanocomposite films. Adv. Funct. Mater. 25, 4328–4333 (2015).

[b19] Schweiger S., Kubicek M., Messerschmitt F., Murer C. & Rupp J. L. M. A microdot multilayer oxide device: let us tune the strain-ionic transport interaction. ACS Nano 8, 5032–5048 (2014). 24720562 10.1021/nn501128y

[b20] Pergolesi D. . Tensile lattice distortion does not affect oxygen transport in yttria-stabilized zirconia-CeO2 heterointerfaces. ACS Nano 6, 10524–10534 (2012). 23106091 10.1021/nn302812m

[b21] Sanna S. . Enhancement of ionic conductivity in Sm-doped ceria/yttria-stabilized zirconia heteroepitaxial structures. Small 6, 1863–1867 (2010). 20690134 10.1002/smll.200902348

[b22] Shen W. D., Jiang J. & Hertz J. L. Beneficial lattice strain in heterogeneously doped ceria. J. Phys. Chem. C 118, 22904–22912 (2014).

[b23] Schichtel N., Korte C., Hesse D. & Janek J. Elastic strain at interfaces and its influence on ionic conductivity in nanoscaled solid electrolyte thin films-theoretical considerations and experimental studies. Phys. Chem. Chem. Phys. 11, 3043–3048 (2009). 19370197 10.1039/b900148d

[b24] Shen W. D., Jiang J. & Hertz J. L. Reduced ionic conductivity in biaxially compressed ceria. RSC Adv. 4, 21625–21630 (2014).

[b25] Mayeshiba T. & Morgan D. Strain effects on oxygen migration in perovskites. Phys. Chem. Chem. Phys. 17, 2715–2721 (2015). 25503536 10.1039/c4cp05554c

[b26] Tealdi C. & Mustarelli P. Improving oxygen transport in perovskite-type LaGaO3 solid electrolyte through strain. J. Phys. Chem. C 118, 29574–29582 (2014).

[b27] De Souza R. A., Ramadan A. & Horner S. Modifying the barriers for oxygen-vacancy migration in fluorite-structured CeO2 electrolytes through strain: a computer simulation study. Energ. Environ. Sci. 5, 5445–5453 (2012).

[b28] Ma D. W. . Effect of lattice strain on the oxygen vacancy formation and hydrogen adsorption at CeO_2_(111) surface. Phys. Lett. A 378, 2570–2575 (2014).

[b29] Cai Z. H., Kuru Y., Han J. W., Chen Y. & Yildiz B. Surface electronic structure transitions at high temperature on perovskite oxides: the case of strained La0.8Sr0.2CoO3 thin films. J. Am. Chem. Soc. 133, 17696–17704 (2011). 21913726 10.1021/ja2059445

[b30] Sun L., Marrocchelli D. & Yildiz B. Edge dislocation slows down oxide ion diffusion in doped CeO2 by segregation of charged defects. Nat. Commun. 6, 7294 (2015). 25723877 10.1038/ncomms7294

[b31] Marrocchelli D., Bishop S. R., Tuller H. L. & Yildiz B. Understanding chemical expansion in non-stoichiometric oxides: ceria and zirconia case studies. Adv. Funct. Mater. 22, 1958–1965 (2012).

[b32] Aschauer U., Pfenninger R., Selbach S. M., Grande T. & Spaldin N. A. Strain-controlled oxygen vacancy formation and ordering in CaMnO_3_. Phys. Rev. B 88, 054111 (2013).

[b33] Greenberg M., Wachtel E., Lubomirsky I., Fleig J. & Maier J. Elasticity of solids with a large concentration of point defects. Adv. Funct. Mater. 16, 48–52 (2006).

[b34] Kossoy A., Feldman Y., Wachtel E., Lubomirsky I. & Maier J. Elasticity of solids with a large concentration of point defects II. The chemical strain effect in Ce_0.8_Gd_0.2_O_1.9_. Adv. Funct. Mater. 17, 2393–2398 (2007).

[b35] Kossoy A. . The origin of elastic anomalies in thin films of oxygen deficient ceria, CeO_2-x_. Solid State Ionics 181, 1473–1477 (2010).

[b36] Yildiz B. ‘Stretching' the energy landscape of oxides-effects on electrocatalysis and diffusion. MRS Bull. 39, 147–156 (2014).

[b37] Wen K. C., Lv W. Q. & He W. D. Interfacial lattice-strain effects on improving the overall performance of micro-solid oxide fuel cells. J. Mater. Chem. A 3, 20031–20050 (2015).

[b38] Song K. . Cerium reduction at the interface between ceria and yttria-stabilised zirconia and implications for interfacial oxygen non-stoichiometry. APL Mater. 2, 032104 (2014).

[b39] Aidhy D. S., Liu B., Zhang Y. & Weber W. J. Strain-induced phase and oxygen-vacancy stability in ionic interfaces from first-principles calculations. J. Phys. Chem. C 118, 30139–30144 (2014).

[b40] Donner W. . Epitaxial strain-induced chemical ordering in La_0.5_Sr_0.5_CoO_3-δ_ films on SrTiO3. Chem. Mater. 23, 984–988 (2011).

[b41] Petrie J. R. . Perovskite: strain control of oxygen vacancies in epitaxial strontium cobaltite films. Adv. Funct. Mater. 26, 1563–1563 (2016).

[b42] Matthews J. W. & Blakeslee A. E. Defects in epitaxial multilayers. 1. Misfit dislocations. J. Cryst. Growth 27, 118–125 (1974).

[b43] Shi Y. . Growth of highly strained CeO_2_ ultrathin films. ACS Nano 10, 9938–9947 (2016). 27934073 10.1021/acsnano.6b04081

[b44] Hayashi H. . Thermal expansion coefficient of yttria stabilized zirconia for various yttria contents. Solid State Ionics 176, 613–619 (2005).

[b45] Hayashi H. . Thermal expansion of Gd-doped ceria and reduced ceria. Solid State Ionics 132, 227–233 (2000).

[b46] Feng Z. L. A., El Gabaly F., Ye X. F., Shen Z. X. & Chueh W. C. Fast vacancy-mediated oxygen ion incorporation across the ceria-gas electrochemical interface. Nat. Commun. 5, 5374 (2014). 25007038 10.1038/ncomms5374

[b47] Chueh W. C. . Highly enhanced concentration and stability of reactive Ce^3+^ on doped CeO2 surface revealed in operando. Chem. Mater. 24, 1876–1882 (2012).

[b48] Balaji Gopal C., El Gabaly F., McDaniel A. H. & Chueh W. C. Origin and tunability of unusually large surface capacitance in doped cerium oxide studied by ambient pressure X-ray photoelectron spectroscopy. Adv. Mater. 28, 4692–4697 (2016). 27031580 10.1002/adma.201506333

[b49] Hao Y., Yang C. K. & Haile S. M. Ceria-zirconia solid solutions (Ce_1-x_Zr_x_O_2-δ_, x <= 0.2) for solar thermochemical water splitting: a thermodynamic study. Chem. Mater. 26, 6073–6082 (2014).

[b50] Dutta G. . Origin of enhanced reducibility/oxygen storage capacity of Ce_1-x_Ti_x_O_2_ compared to CeO_2_ or TiO_2_. Chem. Mater. 18, 3249–3256 (2006).

[b51] Fabris S., de Gironcoli S., Baroni S., Vicario G. & Balducci G. Taming multiple valency with density functionals: a case study of defective ceria. Phy. Rev. B 72, 041102 (2005).

[b52] Grieshammer S., Zacherle T. & Martin M. Entropies of defect formation in ceria from first principles. Phys. Chem. Chem. Phys. 15, 15935–15942 (2013). 23955537 10.1039/c3cp51913a

[b53] Gopal C. B. & van de Walle A. Ab initio thermodynamics of intrinsic oxygen vacancies in ceria. Phys. Rev. B 86, 134117 (2012).

[b54] Ogletree D. F., Bluhm H., Hebenstreit E. D. & Salmeron M. Photoelectron spectroscopy under ambient pressure and temperature conditions. Nucl. Instrum. Meth. A 601, 151–160 (2009).

[b55] Whaley J. A. . Note: fixture for characterizing electrochemical devices in-operando in traditional vacuum systems. Rev. Sci. Instrum. 81, 086104 (2010). 20815633 10.1063/1.3479384

[b56] Dudarev S. L., Botton G. A., Savrasov S. Y., Humphreys C. J. & Sutton A. P. Electron-energy-loss spectra and the structural stability of nickel oxide: an LSDA+U study. Phys. Rev. B 57, 1505–1509 (1998).

[b57] Farra R. . Promoted ceria: a structural, catalytic, and computational study. ACS Catal. 3, 2256–2268 (2013).

[b58] Da Silva J. L. F., Ganduglia-Pirovano M. V., Sauer J., Bayer V. & Kresse G. Hybrid functionals applied to rare-earth oxides: the example of ceria. Phys. Rev. B 75, 045121 (2007).

[b59] Nolan M., Parker S. C. & Watson G. W. The electronic structure of oxygen vacancy defects at the low index surfaces of ceria. Surf. Sci. 595, 223–232 (2005).

[b60] Capdevila-Cortada M., García-Melchor M. & López N. Unraveling the structure sensitivity in methanol conversion on CeO2: A DFT + U study. J. Catal. 327, 58–64 (2015).

[b61] Togo A., Oba F. & Tanaka I. First-principles calculations of the ferroelastic transition between rutile-type and CaCl_2_-type SiO_2_ at high pressures. Phys. Rev. B 78, 134106 (2008).

