# Supplementary material for: Equilibrium oxygen storage capacity of ultrathin CeO2-δ depends non-monotonically on large biaxial strain
Source: Nat Commun. 2017 May 18;8:15360. doi: 10.1038/ncomms15360 (PMC5454370; doi:10.1038/ncomms15360)
Supplement: Supplementary Information — Supplementary Figures, Supplementary Tables, Supplementary Notes and Supplementary References [file ncomms15360-s1.pdf]

# Supplementary Figures

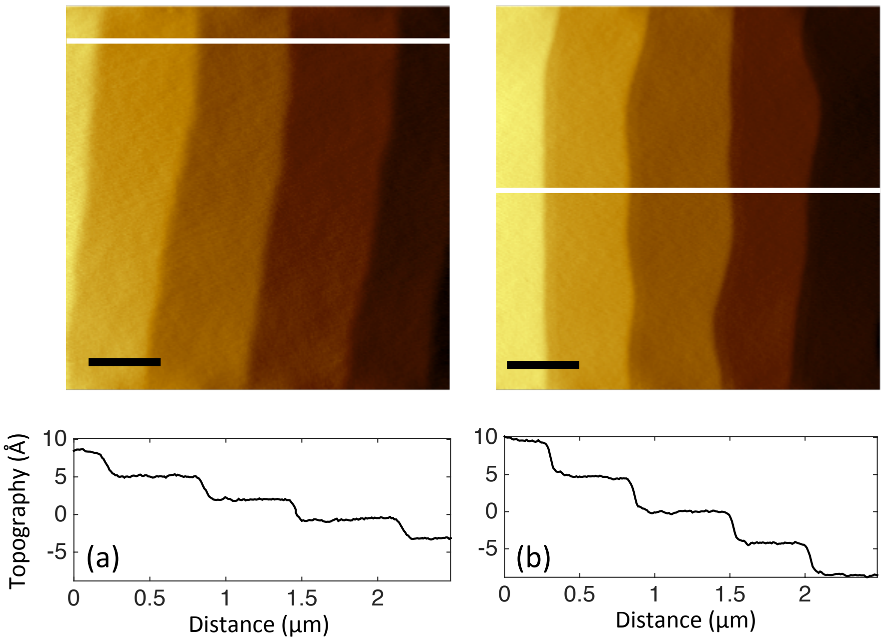


**Supplementary Figure 1 | AFM images of 3 nm ceria films**. Grown on atomically flat (a) YSZ and (b) STO single crystal substrates taken after the APXPS measurements. Both films conform to the terrace-and-step structure of the substrate with step heights of 0.25 nm on YSZ (1/2 unit cell) and 0.4 nm on STO (1 unit cell). Scale bars = 500 nm.

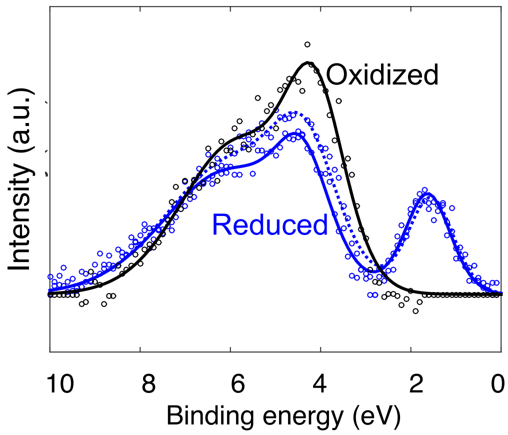

**Supplementary Figure 2 | Reversibility of the redox behavior of the ultrathin ceria films**. Examined by switching from a 55 mTorr mixture of 1 $H_2$:10 $H_2O$ (reducing atmosphere, solid blue line) to 100 mTorr $O_2$ (black line) and back to the initial reducing atmosphere (dashed blue line), at a temperature of 450 ˚C. As illustrated in the VB spectra of the 3 nm ceria film on YSZ, the two blue curves are identical, confirming that the redox process is reversible.

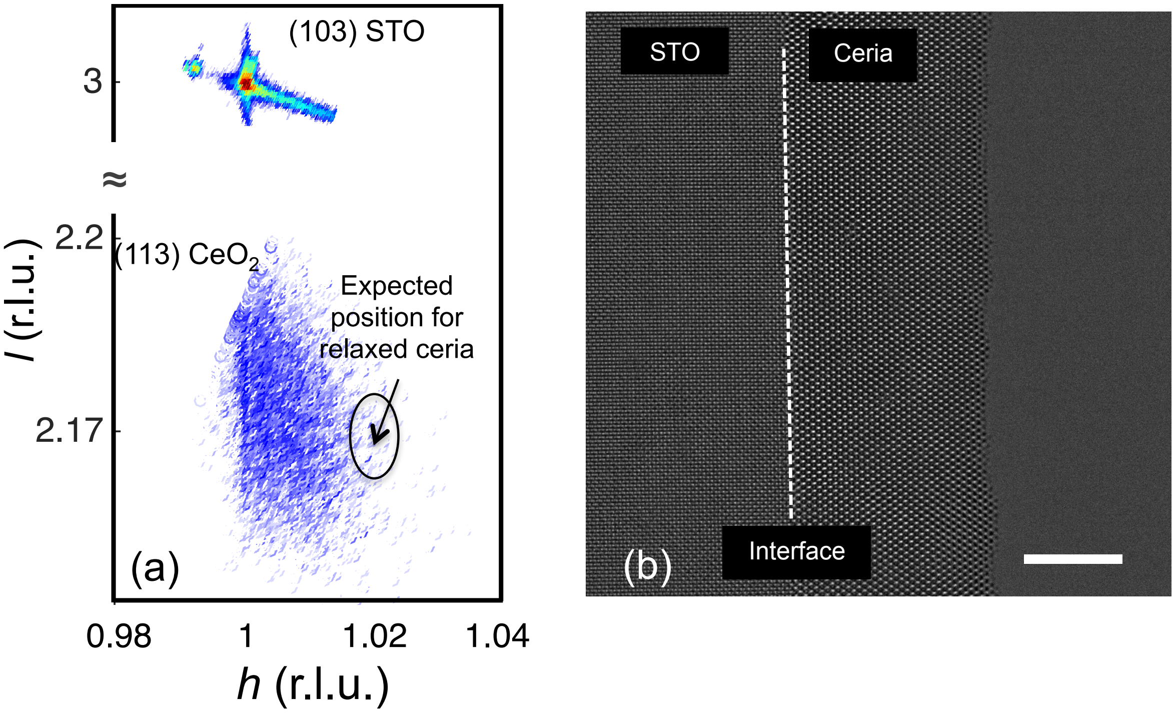


**Supplementary Figure 3 |** (a) X-ray RSM of the 9 nm ceria film on STO about the (103) reflection of the substrate. (b) HR-TEM image reveals a misfit dislocation-free interface. Scale bar = 5 nm.

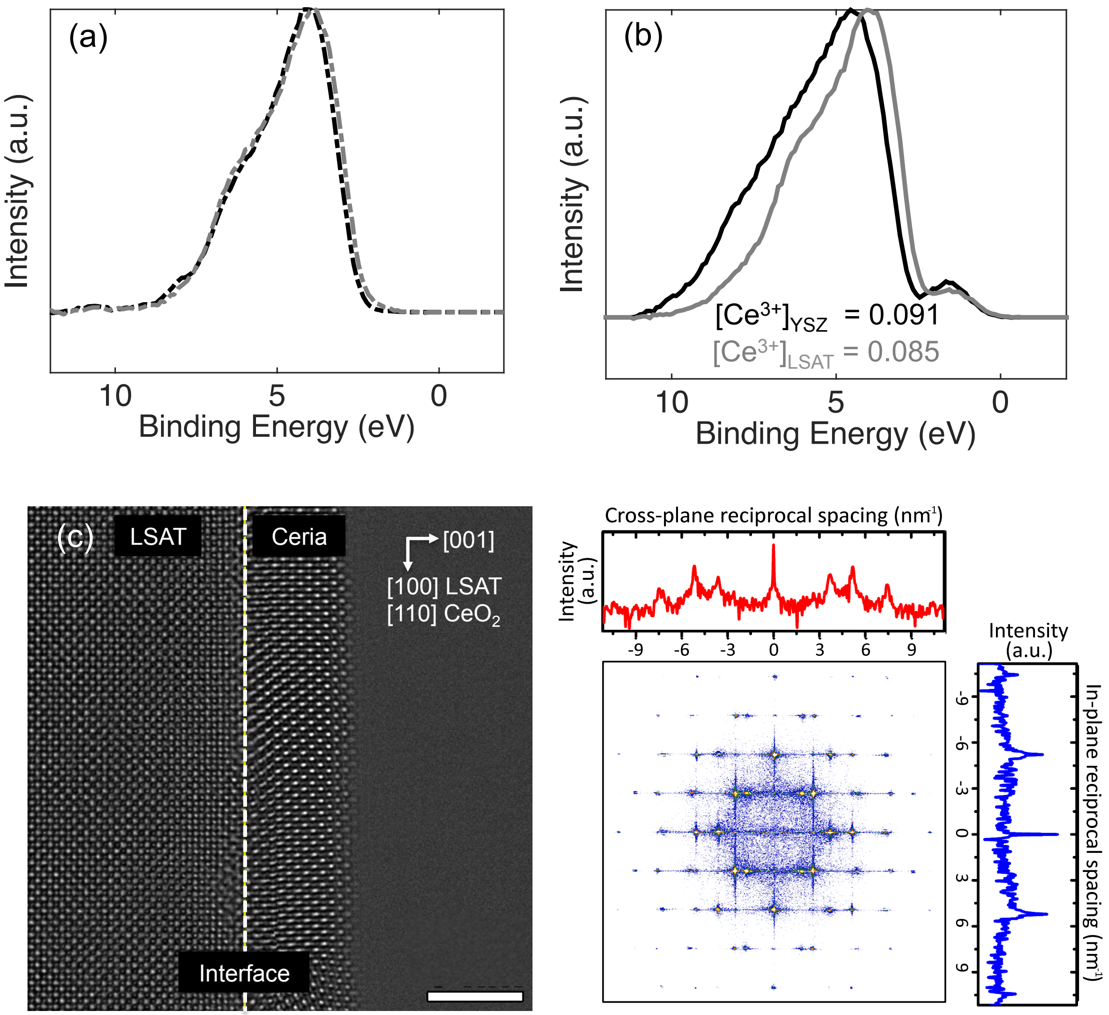


**Supplementary Figure 4 | TEM and XPS of CeO2/LSAT.** Valence-band spectra of 5 nm ceria/LSAT (grey) and 350 nm ceria/YSZ (black) at (a) $pO_2 = 1.3 \times 10^{-4}$ atm, T = 450 ˚C and (b) $pO_2 = \sim 10^{-23}$ atm , T = 450 ˚C. The extent of reduction at the surface is not significantly impacted by the film thickness, so long as the strain states are comparable. (c) HR-TEM image of 2.5 nm $CeO_{2-\delta}$/LSAT. No misfit dislocations were found. FFT patterns of $CeO_{2-\delta}$/LSAT obtained from the corresponding TEM image. Line cuts along the in-plane direction (blue curve) show singlet peaks, further confirming coherency with the substrate. Along the

cross-plane direction (red curve), doublet peaks are observed. Scale bar = 3 nm.

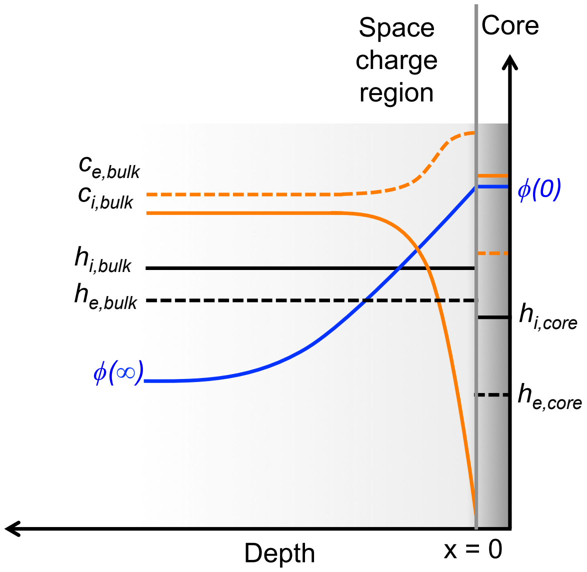


**Supplementary Figure 5 | Simulated formation energy and resulting electric potential distribution profiles.** Schematic of oxygen vacancy and electron formation energy profiles assumed in the formulation of a thermodynamically consistent SC model, and the corresponding concentration and electrostatic potential distributions (not to scale).

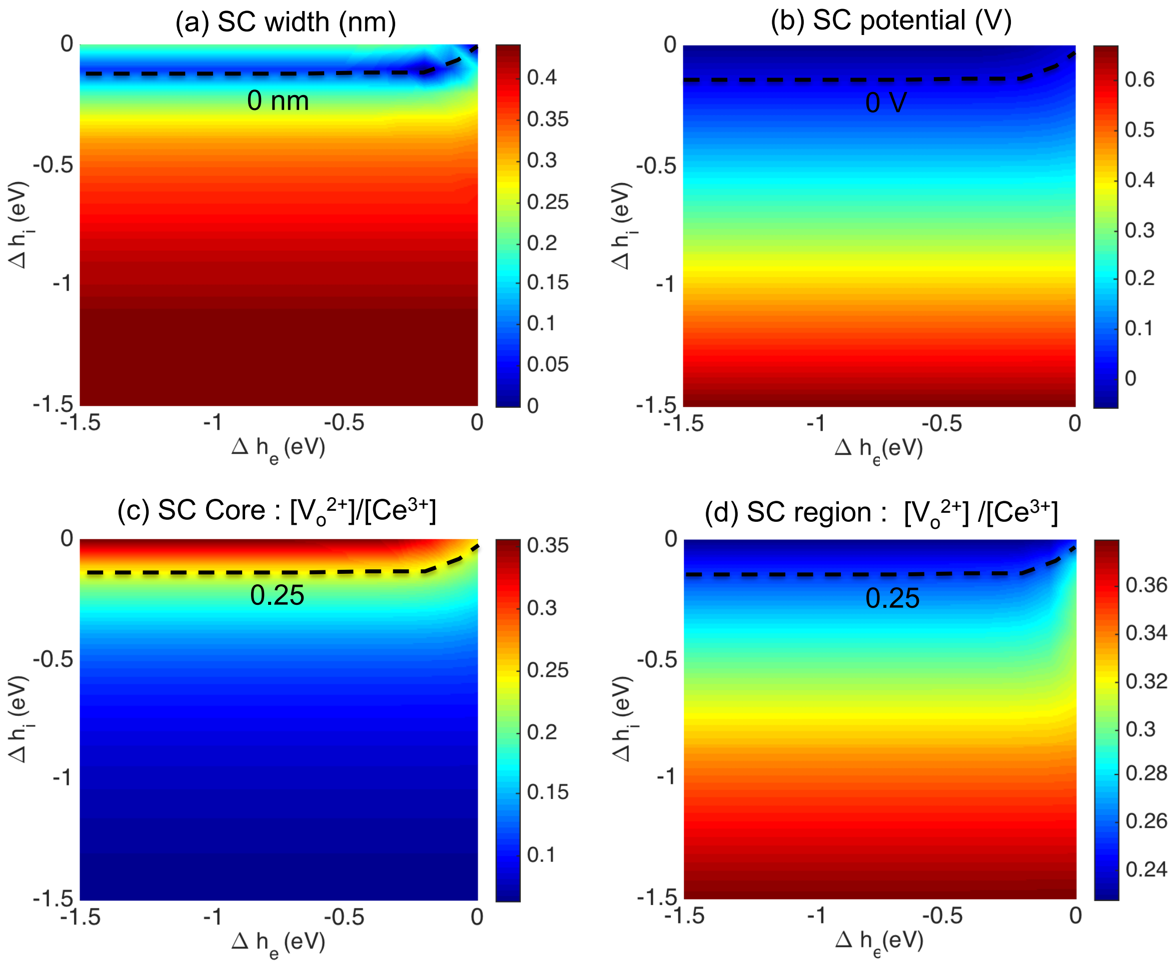


**Supplementary Figure 6 | Simulations of space charge effect on XPS concentrations.** Simulated a) SC width, b) SC core potential, c) ratio of fractional concentration of vacancies to that of electrons in the SC core and d) ratio of fractional concentration of vacancies to that of electrons in the SC region for a range of electron and ion segregation energies. Bulk $\delta = 0.1$ and $T = 773$ K. Color bars indicate values of SC widths in nm, SC core potential in volts, and concentration ratios in dimensionless units. Dashed contours correspond to pairs of electron and ion segregation energies that yield zero space charge potential. Oxygen vacancy fraction is defined relative to the oxygen sublattice, and electron fraction is defined relative

to the Ce sublattice.

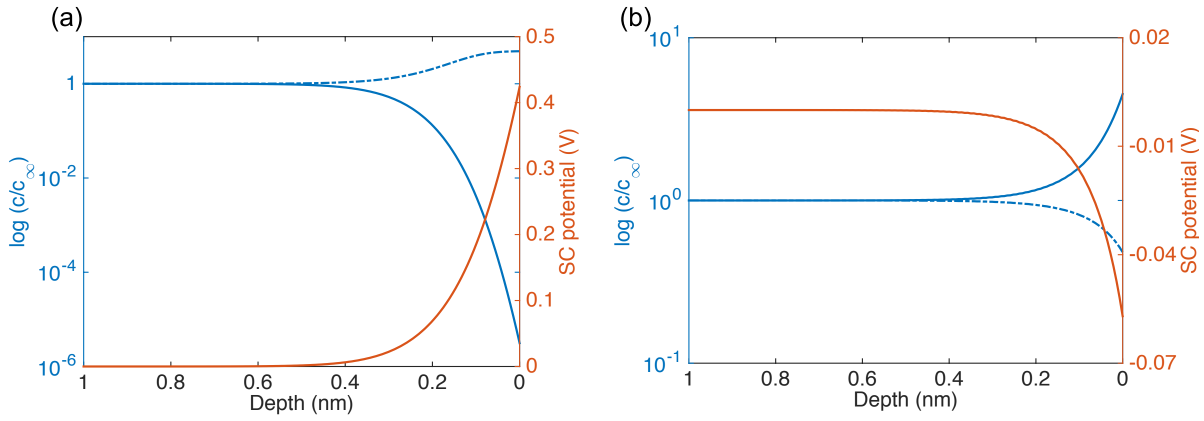


**Supplementary Figure 7 | Defect concentrations for positive and negative space charge potentials.** Simulated distribution of electrostatic potential, electron concentration (dashed) and oxygen vacancy concentration (solid) relative to the bulk in the space charge region for δ = 0.1, T = 773 K. (a) For $\Delta h_i$ = -1 eV and $\Delta h_e$ = -1 eV, a positive SC core potential and an electron accumulation zone is observed in the SC region. (b) For $\Delta h_i$ = 0 eV and $\Delta h_e$ = -1.5 eV, vacancies accumulate in the SC region, and the SC core potential is negative.

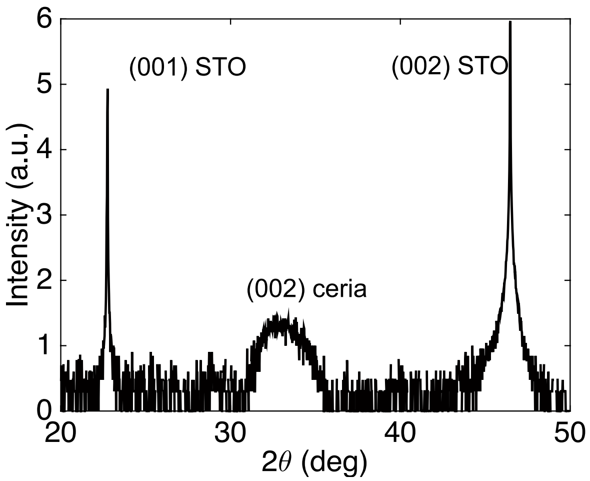

**Supplementary Figure 8 | Out of plane X-ray diffraction scans**. ω-2θ scan of the 3 nm ceria/STO, showing only the (00L) peaks of the substrate and the film. Using the FWHM of the film peak ~ 3.5˚, the thickness of the film is calculated to be ~ 3 nm, in agreement with X-ray reflectivity measurements.

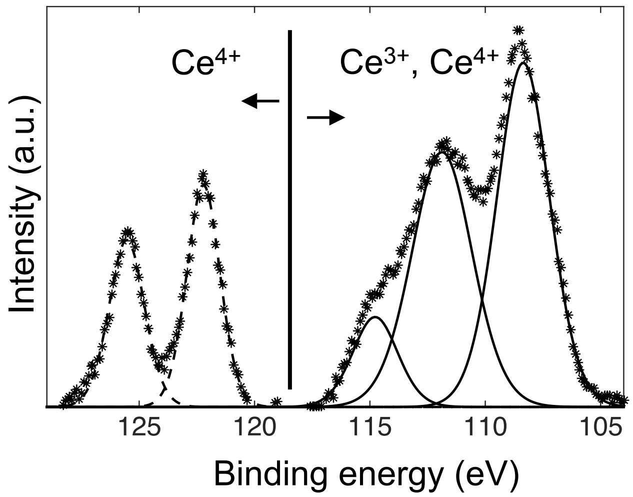


**Supplementary Figure 9 | [$Ce^{3+}$] Quantification using Ce 4d core level spectra**. The two peaks at higher binding energies (dashed lines) arise from spin orbit coupling in $Ce^{4+}$, while the lower binding energy peaks (solid lines) are attributed to XPS final state effects in both $Ce^{4+}$ and $Ce^{3+}$.

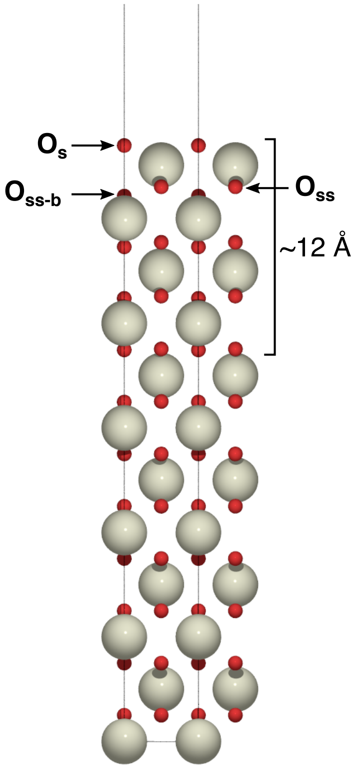


**Supplementary Figure 10 | Simulation cell for $CeO_2$ (100) surface calculations.** Color code: Ce (pale yellow), O (red). Oxygen vacancy formation energies were computed for various sites at increasing depths from the surface ($O_s$, $O_{ss}$, $O_{ss-b}$, $O_{ss-2b}$) within the XPS probing depth (1.2 nm).

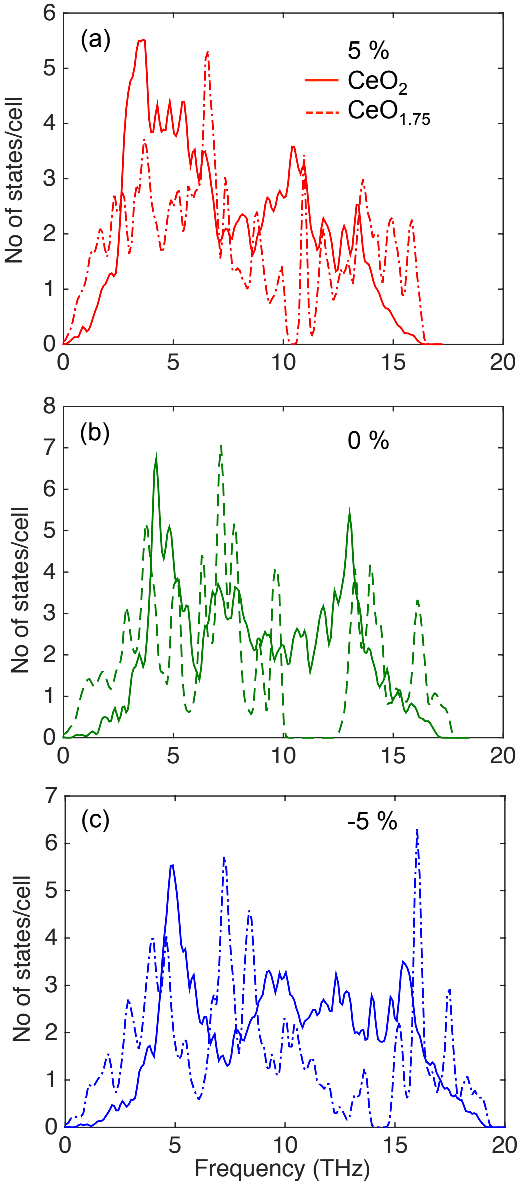


**Supplementary Figure 11 | Effect of biaxial strain on phonon density of states.** Harmonic phonon density of states for a stoichiometric unit cell (solid line) and a nonstoichiometric unit cell (1 O vacancy, dashed line) under a) 5 % biaxial tension, b) no strain, and c) 5 % biaxial compression.

## Supplementary Tables

**Supplementary Table 1 | Quantification for Figure 3d.** Oxygen contents are determined by normalizing the O2p intensity to the fully oxidized internal reference. Analysis using O 1s yielded similar results, although with a slightly greater scatter. The O content decreases as $Ce^{3+}$ content increases, consistent with an approximately electroneutral film. We note that quantification of oxygen vacancy concentration from XPS is semi-quantitative, due to the hybridization of O 2p with Ce 5d and 4f in the final state of valence band spectra.

| **IMFP = 1.2 nm** | | **IMFP = 0.6 nm** | | |
|---|---|---|---|---|
| **[$Ce^{3+}$]** | **[$O^{2-}$]** | **[$Ce^{3+}$]** | **[$O^{2-}$]** | |
| 0.00 | 1.00 | 0.00 | 1.00 | **Ceria/STO** |
| 0.17 | 0.93 | 0.28 | 0.88 | |
| 0.28 | 0.93 | 0.23 | 0.88 | |
| 0.13 | 0.94 | 0.31 | 0.87 | |
| 0.32 | 0.90 | 0.50 | 0.82 | |
| 0.25 | 0.92 | 0.58 | 0.83 | |
| 0.40 | 0.87 | 0.52 | 0.80 | |

| | | | | |
|---|---|---|---|---|
| 0.30 | 0.91 | 0.52 | 0.83 | |
| | | | | |
| 0.03 | 0.98 | 0.24 | 0.93 | |
| 0.27 | 0.90 | 0.49 | 0.83 | |
| 0.06 | 0.97 | 0.41 | 0.87 | **Ceria/YSZ** |
| 0.31 | 0.89 | 0.29 | 0.88 | |
| 0.10 | 1.00 | 0.43 | 0.87 | |

**Supplementary Table 2 | Summary of DFT calculations of vacancy formation energies in 2×2×2 supercells and unit cell of $CeO_{2-\delta}$.** $E_{vac,bi}$ and $E_{vac,iso}$ denote the oxygen vacancy formation energies under biaxial strain and isostatic strain.

| | | **1 vacancy in 2×2×2 supercell (δ=0.03)** | | **2 vacancies in 1×1×1 unit cell (δ=0.50)** |
|---|---|---|---|---|
| **Biaxial Strain** | **Isostatic strain** | $E_{vac,bi}$ **(eV)** | $E_{vac, iso}$ **(eV)** | $E_{vac, bi}$ **(eV)** |
| -5.00% | -2.20% | 3.14 | 3.33 | 3.34 |
| -2.50% | -1.10% | 3.06 | 3.13 | 3.36 |
| 0.00% | 0.00% | 2.89 | 2.89 | 3.26 |

| | | | | |
|---|---|---|---|---|
| 2.50% | 1.10% | 2.56 | 2.58 | 3.07 |
| 5.00% | 2.20% | 1.98 | 2.22 | 2.70 |

**Supplementary Table 3 | DFT calculations of oxygen vacancy formation energies for (100) terminated ceria surface**. 'Position' denotes the most stable oxygen vacancy position for the particular strain state (see Supplementary Figure 10 for reference).

| Biaxial Strain | $E_{vac}$ (eV) | Position |
|---|---|---|
| -5.00 % | 2.71 | Oss-b |
| 0.00 % | 2.52 | Os |
| 5.00 % | 1.59 | Os |

**Supplementary Table 4 | Lattice expansion in a unit cell of bulk ceria per oxygen released**. $a_0$ and a denote optimized cross-plane lattice constants of the stoichiometric ($CeO_2$) and nonstoichiometric ($CeO_{1.75}$) unit cells respectively.

| Strain | 100*(a-$a_0$)/$a_0$ |
|---|---|
| -5.00 % | 4.44 |
| 0.00 % | 3.68 |
| 5.00 % | 3.48 |

**Supplementary Table 5 | Parameters used to compute effective attenuation length of photoelectrons.**

| **Composition** | $CeO_2$ |
|---|---|
| **Density** | 7.1 g $cm^{-3}$ |
| **Number of valence electrons** | 20.4 |
| **Asymmetry parameters** | 0.770 |
| **Band gap** | 4 eV (O 2p - Ce 4f) |
| **X-ray incidence angle** | 15˚ |
| **Electron emission angle** | -40˚ |

**Supplementary Note 1**

## Critical thickness for coherent films

The equilibrium theory of dislocations[1] expresses the critical thickness for a commensurate film, $h_c$, as a function of the strain energy and the barrier for nucleating misfit dislocations. The critical thickness is given by

$$\ln(\alpha h_c / b) = 8\pi(1+\nu)\epsilon(h_c / b) \quad (1)$$

where $\alpha$ is a dimensionless constant typically between 1 and 10 (related to the strength of the dislocation core), $b$ is the magnitude of the Burger's vector, ε is the misfit strain between the film and the substrate and ν is the Poisson's ratio for the film. Solving equation (1) iteratively, it can be shown that $h_c$ is approximately 0.8 nm for α=10. Smaller α values give even smaller critical thicknesses. As a consequence of the large mismatch between YSZ and ceria, equilibrium theory predicts that a coherent film is unlikely.

**Supplementary Note 2**

## Normalization of O 2p feature in the valence band spectra

A semi-quantitative measure of the oxygen stoichiometry is obtained using the O 2p feature in the valence-band (VB) spectra. The area of O 2p feature, $A$(O 2p), is first normalized by the integrated intensity of Ce 4d spectra, $A$(Ce 4d), at identical information depth. This is done to account for fluctuations in beam intensity under different gas conditions. The ratio of this quantity, normalized by that of the oxidized reference is related to the fractional oxygen content near the surface:

$$[O^{2-}]^{\text{semi-quant}} = A(\text{O } 2p)_{norm} = \frac{A(\text{O } 2p)/A(Ce \text{ 4d})}{A(\text{O } 2p)_{\text{ref}} / A(\text{Ce 4d})_{\text{ref}}} \tag{3}$$

Unlike the Ce 4d spectra, the O 2p feature lacks an internal normalization factor. Also, there is partial hybridization between the O 2p and Ce 4f / 5d states. As a result, we note that these ratios are not a fully quantitative measure of oxygen concentration. We also performed a similar determination using the lattice O 1s peak, which yielded a similar result as that shown in Figure 3.

**Supplementary Note 3**

**Quantification of [$Ce^{3+}$]**

For completeness we briefly describe the [$Ce^{3+}$] quantification procedure which has been reported in the work of Chueh *et al.*[2], Feng *et al.*[3] and Balaji Gopal *et al*[4]. The Ce 4d spectra (Supplementary Figure 9) comprise of two sets of peaks. The two higher binding energy (BE) peaks (120-130 eV) are due to $Ce^{4+}$ only, and are split by spin-orbit coupling. The lower BE peaks arise due to $Ce^{3+}$ and $Ce^{4+}$ and are subject to final state effects. Under oxidizing conditions, all the cerium atoms are in the 4+ oxidation state, and the spectrum under this condition serves as a natural reference. Using the reference, the [$Ce^{3+}$] under any condition is calculated as:

$$[\text{Ce}^{3+}] = \frac{A(\text{Ce}^{4+})/A(\text{Ce 4d})}{A(\text{Ce}^{4+})_{\text{ref}} / A(\text{Ce 4d})_{\text{ref}}} \tag{2}$$

where $A(\text{Ce}^{4+})$ represents the area under the higher BE peaks, and $A$(Ce 4d) represents the integrated area of the entire Ce 4d spectra. Subscript 'ref' denotes the oxidized reference.

Additionally, the Ce 4f peak in the valence band spectra (VB) is also proportional to

the concentration of $Ce^{3+}$. The area of Ce 4f peak normalized by the corresponding Ce 4d intensity is a linear function of $[Ce^{3+}]$ (equation (2)). The slope provides a scaling factor to directly calculate $[Ce^{3+}]$ from the VB spectra. In this work, we use Ce 4f spectra for quantifying $[Ce^{3+}]$ due to the smaller scatter in the data.

**Supplementary Note 4**

**Relating equilibrium oxygen vacancy concentration and vacancy formation energy**

For the reduction of ceria at a fixed temperature, the redox reaction is given by

$$\mathrm{O_O^{\times}} + 2\mathrm{Ce_{Ce}^{\times}} \rightarrow \mathrm{V_O^{\bullet\bullet}} + 2\mathrm{Ce_{Ce}^{\prime}} + \tfrac{1}{2}\mathrm{O_2} \tag{13}$$

The oxygen chemical potential in the gas phase, fixed by $pO_2$, must equal that in the $CeO_{2-\delta}$ films under equilibrium. The standard enthalpy, $\Delta H_r^0$ and entropy of reduction, $\Delta S_r^0$ are related to the activities of points defects ('$a$') through the law of mass action,

$$\exp\left(-\frac{\Delta H_r^0 - T\Delta S_r^0}{kT}\right) = K_r = \frac{a_{\mathrm{Vac}} a_{Ce^{3+}}^2}{a_{\mathrm{O}^{2-}} a_{Ce^{4+}}^2} p\mathrm{O}_2^{1/2} \tag{14}$$

where $K_r$ is the equilibrium constant. Application of strain shifts this equilibrium by modifying the energetics of oxygen vacancy formation. Specifically, we observe up to 4-fold enhancement in the oxygen nonstoichiometry under strain. Using the $[Ce^{3+}]$ measured by APXPS, an estimate of the change in reduction enthalpy can be obtained conditional on a few assumptions. First, we do not consider defect-defect interaction in estimating the enthalpy as we do not have a clear picture of how such interaction depends on strain. Accordingly, we express equation (14) in terms of $\delta$, the oxygen nonstoichiometry in $CeO_{2-\delta}$:

$$K_r = \frac{(2\delta)^2(\delta/2)}{(1-2\delta)^2(1-\delta/2)} pO_2^{1/2} \tag{15}$$

Second, we approximate $\Delta H_r^0$ as invariant with δ (in reality, bulk values vary by 20 % with δ[5]). Finally, we assume non-configurational contributions to the solid-state entropy is not dependent on strain. Using subscripts "*str*" and "*rel*" to denote the strained and relaxed oxides, and eliminating $pO_2$ from equation (15), we get,

$$\Delta H_{r,\,\mathrm{str}}^0 - \Delta H_{r,\,\mathrm{rel}}^0 = kT \ln\left( \frac{(2\delta_{rel})^2(\delta_{rel}/2)}{(1-2\delta_{rel})^2(1-\delta_{rel}/2)} * \frac{(1-2\delta_{str})^2(1-\delta_{str}/2)}{(2\delta_{str})^2(\delta_{str}/2)} \right) \tag{16}$$

By substituting for $\delta_{rel}$ and $\delta_{str}$ from Figure 4d,e we estimate that the enthalpy of oxygen vacancy formation for the strained films can be as much as 0.4 eV lower than that for the relaxed films.

**Supplementary Note 5**

**Redox behavior of ultrathin ceria films on $La_{0.18}Sr_{0.82}Al_{0.49}Ta_{0.41}O_3$ (LSAT)**

APXPS measurements were also performed on an ultrathin ceria film grown on atomically flat (001) LSAT substrate (MTI). LSAT, with a lattice constant of 3.868 Å, has the lowest lattice mismatch with ceria (~ 0.7 %) of the readily available cubic structured oxides, and is also stable under reducing conditions used in this work. LSAT substrates were annealed at 1200 ˚C in 21 % $O_2$ for 8 h to obtain step terraces. TEM images revealed the absence of misfit dislocations, and in-plane and cross-plane lattice constants of the ceria film were 5.461 Å and 5.406 Å respectively.

Both core level and VB spectra were collected using photoelectrons with KE = 250 eV and

690 eV for the two films under oxidizing and reducing conditions. The spectra of the two samples are quantitatively similar (with [$Ce^{3+}$] difference less than 10 %), justifying our choice of the truly unstrained, thick film as the reference sample (Supplementary Figure 4).

**Supplementary Note 6**

**Effect of space charge on defect concentrations measured by photoemission**

**Thermodynamics of SC formation**

A detailed treatment of the effect of space charge potential on photoemission spectra can be found in the work of Shavorskiy *et al*[6] . Equilibrium SC zones result from segregation of charged defects from the bulk to interfaces or surfaces. This segregation of charged defects sets up an electrostatic potential gradient to equilibrate the electrochemical potential across the entire sample. The extent of defect accumulation and depletion, and the resulting potential are a function of this driving force, mobile defect concentrations, dielectric constant, etc. We perform a thermodynamically-consistent SC analysis by independently considering electrons and oxygen vacancy segregation from the bulk to the surface core, building up on previous work in the literature[7].

In this model, we assume that defect formation energies change abruptly at the interface of a thin surface region ("surface core") and the rest of the solid (position x = 0, Supplementary Figure 5). We denote the segregation energies for ions (oxygen vacancies) and electrons as $\Delta h_i = h_{core,i} - h_{bulk,i}$ and $\Delta h_e = h_{core,e} - h_{bulk,e}$, respectively. In this model, we do not consider other sources of charge, such as those due to adsorbates and the additional SC zones near the film/substrate interface. Equilibration of electrochemical potential between

the bulk and core requires that

$$\Delta h_i + kT\ln\left(\frac{c_{core,\,i}}{N_i - c_{core,\,i}}\right) - kT\ln\left(\frac{c_{bulk,\,i}}{N_i - c_{bulk,\,i}}\right) + z_i e\Phi_0 = 0$$

$$\Delta h_e + kT\ln\left(\frac{c_{core,\,e}}{N_e - c_{core,\,e}}\right) - kT\ln\left(\frac{c_{bulk,\,e}}{N_\mathrm{e} - c_{bulk,\,e}}\right) + z_e e\Phi_0 = 0 \qquad (4)$$

where subscripts *i* and *e* denote oxygen vacancies and electrons respectively, $c_\mathrm{i}$ and $c_e$ denote oxygen vacancy and electron ($Ce^{3+}$) concentrations, $N_i$ and $N_e$ denote the respective site concentrations, (such that $c/N$ is the fractional concentration), $z$ denotes the charge and $\Phi_0$ denotes the potential at x = 0 referenced to the bulk ("SC potential"). $N$ is taken to be identical in the bulk and core. The configurational entropy terms assume an ideal, non-dilute solid solution. For simplicity, we also assume that the thickness of the core is sufficiently thin such that we can ignore the electrostatic potential variation within the core.

For non-zero defect segregation energies, a SC potential ($\Phi_0$) is generated at the surface core. In the SC region adjacent to the core, charge carriers redistribute to screen the potential build-up at the core, and the electrostatic potential gradually decays to the bulk value away from the core. The electrostatic potential and concentrations are related by the Poisson equation as:

$$\epsilon_0\epsilon_r \frac{\partial^2\phi}{\partial x^2} = -\rho(x) \qquad (5)$$

The charge density is given by

$$\rho(x) = e(z_i c_i(x) + z_e c_e(x)) \qquad (6)$$

The individual defect concentrations can be written in a similar fashion as Eq. (4), giving

$$\rho(x)=e\left(\frac{z_i N_{bulk,i}}{1+\frac{N_{bulk,i}-c_{bulk,i}}{c_{bulk,i}}\exp\left(\frac{ez_i\phi(x)}{kT}\right)}+\frac{z_e N_{bulk,e}}{1+\frac{N_{bulk,e}-c_{bulk,e}}{c_{bulk,e}}\exp\left(\frac{ez_e\phi(x)}{kT}\right)}\right) \quad (7)$$

The boundary conditions are:

a. Zero electrostatic potential gradient far from the x= 0 interface:

$$\phi((n-1)\Delta x)=\phi(n\Delta x) \quad (8)$$

b. Matching the core charge ($Q_{c,eq}$) calculated by Gauss law to the core charge determined from the segregation thermodynamics:

$$-2\epsilon_r\epsilon_0\left(\frac{\partial\phi}{\partial x}\right)_{x=0}=Q_{c,eq}=2ew_c(z_i(c_{core,\,i}-c_{bulk,\,i})+z_e(c_{core,\,\mathrm{e}}-c_{bulk,\,\mathrm{e}})) \quad (9)$$

Using Eq. (4), RHS can be recast in terms of $c_{bulk}$, $\Delta h$ and $\Phi_0$ from the previous iteration. The core width, $w_c$ is taken to be 1 nm (*i.e.*, 2 unit cell lengths of $CeO_2$). This value likely overestimates the core width (typically predicted to be less than one atomic layer by DFT). Nevertheless, choosing a thicker core gives a higher core charge, which favors formation of a SC zone.

Using Eq. (9), $\phi(x=0)$ at j+1 iteration can be calculated as :

$$\phi(x=0)^{j+1}=\frac{Q_{c,\,eq}\Delta x}{2\epsilon_r\epsilon_0}+\phi(\Delta x)^{j+1} \quad (10)$$

To solve the Poisson equation numerically, we use finite differences to compute derivatives,

yielding

$$\frac{(\phi(x+\Delta x)-\phi(x))-(\phi(x)-\phi(x-\Delta x))}{(\Delta x)^2}=-\frac{\rho(x)}{\epsilon_r\epsilon_0} \tag{11}$$

The value of $\phi(x)$ at iteration $j+1$ can be calculated based on values from iteration $j$ as

$$\phi(x)^{j+1}=\frac{\phi(x+\Delta x)^j+\phi(x-\Delta x)^j+(\Delta x)^2\frac{\rho(x)^j}{\epsilon_r\epsilon_0}}{2} \tag{12}$$

Thus, Eq. (8) and (10) are used to calculate the potential at the boundaries, while Eq. (12) updates the interior points. The system of equations is iterated until convergence is achieved.

**Results of SC analysis**

Supplementary Figure 6a,b show heat maps of SC width and core potential as a function of electron and ion segregation energies. The simulations were performed using bulk $\delta = 0.1$ (*i.e.* 20% of the Ce in the 3+ state) and T = 773 K. It is readily apparent that $\Delta h_i$ has a much stronger effect on the SC, compared to $\Delta h_e$. This saturation is explained by the fact that $z_i = +2$ and $z_e = -1$ (causing the electrochemical potential of ions in the core to be more sensitive to $\Phi_0$ than that of electrons) and by the fact that $c_e = 2c_i$ and $N_e = N_i/2$ (causing electrons to exhibit site exclusion more so than ions). The hypothesis was confirmed by running the simulations using identical site concentrations and equal but opposite charges for electrons and ions. The SC width reaches a maximum of 0.45 nm for $\Delta h_i$ values approaching –1.5 eV, corresponding to a core potential of 0.65 V. At small $\Delta h_i$, but large $\Delta h_e$, the SC width exhibits an inflection point (dashed line), as the SC potential switches from a positive

to a negative value. Representative plots of the electrostatic potential and concentration distributions are shown in Supplementary Figure 7 for (a) a positively charged and (b) a negatively charged SC core.

In order to compare the simulation to experimental XPS results, we consider two limits in which the XPS probes (1) only the core region, and (2) only the SC/bulk region. In the first limit in which XPS only measures the core with no influence from the bulk, Supplementary Figure 6c shows $[V_o^{2+}]/[Ce^{3+}]$ ratio in the core. The charge-neutral condition is indicated by a dashed line. Here, the core $[V_o^{2+}]/[Ce^{3+}]$ ratio depends strongly on the segregation energies, and vanishes to zero for large $\Delta h_i$. In the second limit, the concentration profiles of electrons and ions at every ($\Delta h_i$, $\Delta h_e$) were depth-weighted by the Beer-Lambert law to obtain the 'averaged' concentration measured by XPS. Supplementary Fig S6d depicts the $[V_o^{2+}]/[Ce^{3+}]$ ratio for an IMFP = 0.6 nm. The relatively weak effect of SC on $[V_o^{2+}]/[Ce^{3+}]$ (even for large segregation energies) arise from the narrow SC width relative to the probing depth. We also recognize the inclusion of gradient energy by combining SC and phase-field treatments will lead to thicker SC region[8], but at the same time more extreme $[V_o^{2+}]/[Ce^{3+}]$ values measured by XPS.

The XPS measured $[V_o^{2+}]/[Ce^{3+}]$ ratio of 0.3 (Figure 3d) is in slight excess of the expected 0.25 in the electroneutral limit. Based on our simulation, this experimental result corresponds to a $\Phi_0$ of < 0.1 eV (in the limit that XPS only probes the core) and a $\Phi_0$ of ~ 0.2 eV (in the limit that XPS only probes the SC region/bulk). From this comparison, we conclude that (1) if XPS probes mostly the surface core, the experimental $[V_o^{2+}]/[Ce^{3+}]$ ratio suggests that a very weak $\Phi_0$, and (2) if the XPS probes the SC/bulk region, it is likely too

thin to contribute significantly to the observed strain effects, and that the observed behavior correspond primarily to defects in the bulk. Moreover, the fact that the experimental $[V_o^{2+}]/[Ce^{3+}]$ ratio does not depend on misfit strain suggests SC effect does not explain the unusual strain dependence of the defect concentrations we observed.

**Supplementary Note 7**

**XPS information depth**

The IMFP was obtained using the NIST Standard Reference Database 82[9] and the TPP-2M equation[10]. The parameters that were input for this calculation are summarized in Supplementary Table 5 and have been used in previous publications[2, 3]. For inorganic compounds, the IMFP is most sensitive to valence electron density, with an average uncertainty of 11 %[10].

**Supplementary Note 8**

**Treatment of electron localization in DFT +*U* calculations**

For the bulk unit cells with an O vacancy, the structures with the lowest energy have the reduced $Ce^{3+}$ ions along the xy plane, while the unit cells with 2O vacancies have all the Ce atoms in the 3+ oxidation state. All the 2 × 2 × 2 supercell structures have the $Ce^{3+}$ ions in the nearest-neighbor positions from the oxygen vacancy. According to a previous work, the energy difference between this electron localization and the most stable one (i.e. next-nearest-neighbor) is less than 0.05 eV, which is within the intrinsic error of DFT[11]. In the case of the $CeO_2(100)$ surfaces, the structures with the lowest energy have the excess of

charge localized at the Ce atoms of the first and second layers in an antiferromagnetic (AFM) ground state. For comparison, we also investigated the defective surface under compressive strain in a ferromagnetic (FM) spin state, obtaining an energy difference between the AFM and FM structures of less than 5 meV. Hence, the spin ground state of the reduced surfaces has a very small effect, in agreement with previous theoretical studies[12]. Our findings for the unstrained $CeO_2(100)$ surface are also in agreement with previous hybrid DFT calculations[13].

## References


1. Matthews JW, Blakeslee AE. Defects in Epitaxial Multilayers .1. Misfit Dislocations. *J Cryst Growth* 1974, **27**(Dec)**:** 118-125.

2. Chueh WC, McDaniel AH, Grass ME, Hao Y, Jabeen N, Liu Z, *et al.* Highly Enhanced Concentration and Stability of Reactive Ce3+ on Doped CeO2 Surface Revealed In Operando. *Chem Mater* 2012, **24**(10)**:** 1876-1882.

3. Feng ZLA, El Gabaly F, Ye XF, Shen ZX, Chueh WC. Fast vacancy-mediated oxygen ion incorporation across the ceria-gas electrochemical interface. *Nat Commun* 2014, **5:** 5374.

4. Balaji Gopal C, El Gabaly F, McDaniel AH, Chueh WC. Origin and Tunability of Unusually Large Surface Capacitance in Doped Cerium Oxide Studied by Ambient Pressure X-ray Photoelectron Spectroscopy. *Adv Mater* 2016**:** (in print).

5. Panlener RJ, Blumenthal RN, Garnier JE. Thermodynamic Study of Nonstoichiometric Cerium Dioxide. *J Phys Chem Solids* 1975, **36**(11)**:** 1213-1222.

6. Shavorskiy A, Ye X, Karslioglu O, Hartl M, Zegkinoglou I, Trotochaud L, *et al.* Direct mapping of band positions in doped and undoped hematite during photoelectrochemical water splitting. *(In preparation)*.

7. De Souza RA. The formation of equilibrium space-charge zones at grain boundaries in the perovskite oxide SrTiO3. *Phys Chem Chem Phys* 2009, **11**(43)**:** 9939-9969.

8. Mebane DS, De Souza RA. A generalised space-charge theory for extended defects in oxygen-ion conducting electrolytes: from dilute to concentrated solid solutions. *Energ Environ Sci* 2015, **8**(10)**:** 2935-2940.

9. Powell CJ, Jablonksi A. NIST Electron Effective-Attentuation-Length Database Version 1.3, SRD 82;. *National Institute of Standards and Technology: Gaithersburg, MD* 2011.

10. Tanuma S, Powell CJ, Penn DR. Calculations of electron inelastic mean free paths. *Surf Interface Anal* 2005, **37**(1)**:** 1-14.

11. Wang B, Xi X, Cormack AN. Chemical Strain and Point Defect Configurations in Reduced Ceria. *Chem Mater* 2014, **26**(12)**:** 3687-3692.

12. Ganduglia-Pirovano MV, Da Silva JLF, Sauer J. Density-Functional Calculations of the Structure of Near-Surface Oxygen Vacancies and Electron Localization on ${\mathrm{CeO}}_{2}(111)$. *Phys Rev Lett* 2009, **102**(2)**:** 026101.

13. Nolan M. Hybrid density functional theory description of oxygen vacancies in the CeO2 (1 1 0) and (1 0 0) surfaces. *Chem Phys Lett* 2010, **499**(1–3)**:** 126-130.
